# Combinatorial Targeting of Avapritinib-Driven MAP Kinase Activation in High-Grade Glioma

**DOI:** 10.21203/rs.3.rs-9974885/v1

**Published:** 2026-06-24

**Authors:** Kallen Schwark, Kelsey Wink, Antonella De Cola, Madeline Miclea, Tirth Patel, Seongbae Kong, Alina Stanczak, Robert Doherty, Ashley Pearson, Bhuvna Murthy, Benison Lau, Rodrigo T. Cartaxo, Dana Messinger, Sunjong Ji, Jack Wadden, Madison Clausen, Daniel de la Nava, Peyton Goethe, Fareen Momen, Tiffany Adam, Aria Hutchinson, Michael Niculcea, Habib Serhan, Nathan M. Merrill, Amy Edmonds, Lisa Mayr, Sina Neyazi, Denise P. Muñoz, Yeonjoo Hwang, Nicholas K. Nuechterlein, Ester Calvo Fernández, Andrea Califano, Jovana Pavisic, Hugh Garton, Rama Rao, Santhosh A. Upadhyaya, Mary Skrypek, Anne E. Bendel, Mina Lobbous, Darren Klawinski, Sofia D. Merajver, Mateusz P. Koptyra, Adam C. Resnick, Javad Nazarian, Sriram Venneti, Sebastian M. Waszak, Lukas Chavez, Johannes Gojo, Mariella G. Filbin, Alexander Beck, Manav Pathania, Jean-Philippe F Coppe, Carl Koschmann

**Affiliations:** 1 Department of Pediatrics, University of Michigan; Ann Arbor, MI, USA.; 2 Ludwig Institute for Cancer Research; Oxford, United Kingdom.; 3 Department of Neurosurgery, University of Michigan; Ann Arbor, MI, 48109, USA.; 4 Department of Internal Medicine, University of Michigan; Ann Arbor, MI, USA.; 5 Department of Pediatrics and Adolescent Medicine, Medical University of Vienna; Vienna, Austria.; 6 Department of Pediatric Oncology, Dana-Farber/Boston Children’s Cancer and Blood Disorder Center and Harvard Medical School; Boston, MA, USA.; 7 Department of Radiation Oncology, University of California San Francisco; San Francisco, CA, USA.; 8 Department of Pathology, University of Michigan; Ann Arbor, MI, USA.; 9 Department of Systems Biology, Columbia University; New York, NY, USA.; 10 Department of Pediatrics, Columbia University; New York, NY, USA.; 11 Department of Pediatric Hematology-Oncology, Children’s Minnesota; Minneapolis, MN, USA.; 12 Department of Neurology, Cleveland Clinic Lerner College of Medicine of Case Western Reserve University; Cleveland, OH, USA.; 13 Department of Pediatrics, Nemours Children’s Health System; Jacksonville, FL, USA.; 14 Department of Pathology and Laboratory Medicine, Children’s Hospital of Philadelphia; Philadelphia, PA, USA.; 15 Department of Surgery, Children’s Hospital of Philadelphia; Philadelphia, PA, USA.; 16 Department of Pediatrics, University Children’s Hospital, University of Zurich, Zurich, Switzerland.; 17 School of Life Sciences, École Polytechnique Fédérale de Lausanne; Lausanne, Switzerland.; 18 Department of Medicine, University of California San Diego; La Jolla, CA, USA.; 19 Center for Neuropathology, Ludwig Maximillian University of Munich; Munich, Germany.

## Abstract

PDGFRA alterations define a high-risk subset of high-grade glioma (HGG), yet targeted therapies have yielded limited and transient benefit. Here, we show that the CNS-penetrant PDGFRA inhibitor avapritinib induces sustained MAPK pathway activation at supratherapeutic dosing, revealing a therapy-induced adaptive vulnerability. High-dimensional kinome profiling (>900 nodes) and *in vivo* studies demonstrate robust, dose-dependent ERK activation following avapritinib treatment. This response is enriched in cycling oligodendrocyte precursor cell-like (OPC-like) tumor populations and promotes survival through ERK-dependent stabilization of the anti-apoptotic protein MCL-1. Modeling of clinically relevant PDGFRA variants reveals that D842V-mutant tumor cells exhibit heightened MAPK activation and potential for MAPK co-targeting. Rational combination strategies suppress this adaptive signaling, with MEK inhibition producing durable pathway suppression and significant survival benefit *in vivo*. Translation to patients demonstrates feasibility and early clinical activity, including a sustained complete regression in an unresected PDGFRA-mutant HGG treated with avapritinib and the MEK1/2 inhibitor selumetinib. Together, these findings identify adaptive MAPK reactivation as a targetable liability and support combined PDGFRA–MAPK inhibition as a therapeutic strategy.

## Introduction:

High-grade glioma (HGG) is a malignant and invasive brain tumor which is incurable in a vast majority of cases, with less than 10% of patients surviving past 2 years.^1,2^ HGGs undergo advanced molecular testing including tumor sequencing, methylation profiling and, increasingly, detection of cell-free tumor DNA (cf-tDNA) in liquid specimens. Previously, we have demonstrated that molecular characterization of HGGs can result in effective targeted therapies.^3–8^

One of the most frequently altered genes in HGG is Platelet-derived growth receptor alpha (*PDGFRA*), which is activated by hetero- and homo-dimers of the PDGF-A and B ligands.^2,4,9,10^ *PDGFRA* is either mutated or amplified in 12% of adult HGG (aHGG) and 15% of pediatric HGG (pHGG).^4,8,11,12^ We have demonstrated that different *PDGFRA* alterations, such as mutations and/or amplifications, are closely associated with factors like patient age, tumor location, and survival outcomes.^4,5,7,13^

*PDGFRA* mutations lead to ligand-independent receptor activation and oncogenic effects, while *PDGFRA* amplification leads to enhanced ligand-dependent signaling.^8,12,14^ Both alteration types activate the downstream pathways Mitogen-Activated Protein Kinase (MAPK) and PI3K/mTOR pathways, which in turn drive cell growth and differentiation.^8,12^ Mutations in exon 18 of the kinase domain, such as D842V, lock the receptor in an active state.^15^ Previous trials of promising PDGFRα inhibitors for HGG have been unsuccessful.^16–21^ Currently, therapies for *PDGFRA*-driven HGG remain non-specific, with limited long-term survivors.

The PDGFRα inhibitor avapritinib is a selective tyrosine kinase inhibitor (TKI) for both exon 18-mutant PDGFRα and exon 17-mutant variants of the receptor tyrosine kinase KIT. It is currently FDA approved for adult patients with unresectable or metastatic gastrointestinal stromal tumors (GIST) harboring PDGFRA exon 18 mutations, adult patients with indolent systemic mastocytosis (ISM), and adult patients with advanced systemic mastocytosis (AdvSM).^22^ Avapritinib is a type I kinase inhibitor specifically designed to retain high potency against exon 18 mutations by binding to the mutated activation loop in its active state.^15^ We previously showed that avapritinib exhibits enhanced selectivity for PDGFRα compared to dasatinib and previous TKIs in cell-free kinase assays as well as excellent blood-brain barrier (BBB) penetration in mice.^8^ In an aggressive *PDGFRA*-driven HGG in utero electroporation (IUE) mouse model, avapritinib significantly extended survival and markedly reduced brain tumor p-PDGFRα levels.^8^ Further, 3 of 8 patients with HGG treated with avapritinib demonstrated radiographic response.^8^

However, because single-agent trials targeting *PDGFRA*-driven HGG have largely failed,^17^ combinatorial therapy is crucial for effective treatment strategies. Documented mechanisms of resistance to PDGFRα/KIT-targeting TKIs in other cancers include but are not limited to: 1) secondary mutations, as has been documented in TKI-resistant GIST;^23,24^ 2) reflexive increases in downstream pathway activity not completely deactivated by PDGFRA inhibition;^13^ or 3) upregulation of alternative RTKs that maintain the same pathways as the inhibited receptor.^25^ It will be critical to determine downstream signaling avapritinib treatment in HGG and optimal combinatorial options to fully harness the full therapeutic potential. Further, little is known about the impact of avapritinib on different variants of *PDGFRA* alterations in HGG (wildtype amplified vs. external domain mutation vs. kinase domain mutation), limiting the optimization of its use.

Here, we discover that prolonged PDGFRα inhibition with avapritinib in HGG, despite being upstream of MAPK signaling, induces sustained MAPK pathway activation and limits the efficacy of monotherapy. This adaptive response is driven primarily by cycling OPC-like tumor cells and promotes survival through ERK-dependent upregulation of MCL-1. Combined PDGFRα and MAPK pathway inhibition overcomes this resistance, supporting dual targeting as a promising therapeutic strategy.

## Results

### Kinome profiling in HGG models to identify potential combinatorial partners of avapritinib

**Figure 1:**
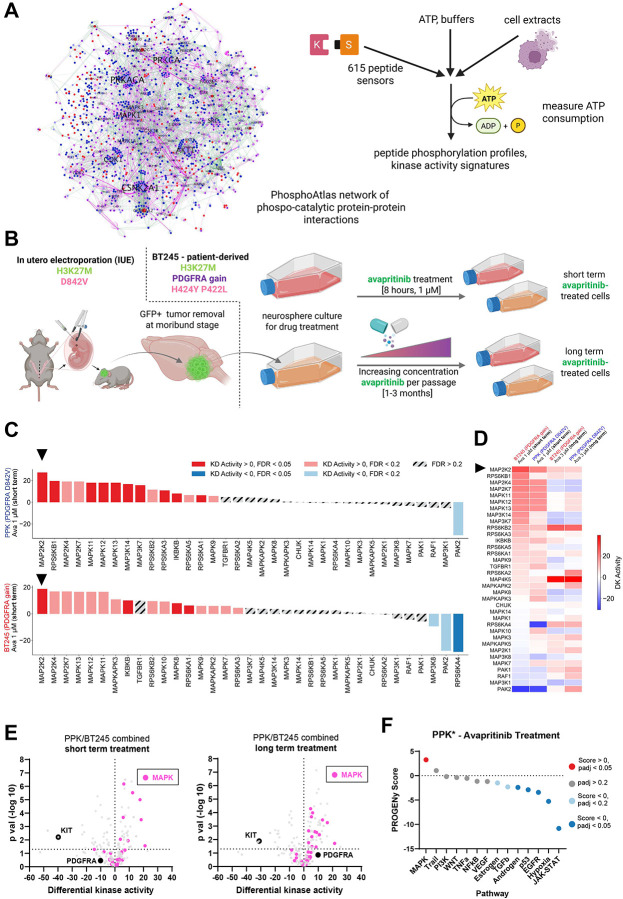
Kinome profiling in HGG models to identify potential combinatorial partners of avapritinib (**A**) Schema explaining high throughput kinase activity mapping (HT-KAM). (**B**) Schema demonstrating generation of avapritinib-resistant cell models from in utero electroporation (PPK, PPK*) or derived from patient (BT245). (**C**) Results from HT-KAM assay after short-term and long-term avapritinib exposure in both PPK and BT245 models, highlighting proteins in the MAPK pathway and ordered by differential kinase activity. (**D**) Heat map of HT-KAM results. (**E**) Results from HT-KAM, in volcano plot form. (**F**) GSEA data from avapritinib-resistant vs. parental PPK* cells, using the PROGENy tool.

Given that PDGFRA is a receptor tyrosine kinase (RTK) embedded within a highly interconnected signaling network, we sought to define global kinase activity changes following its inhibition. To capture pathway-wide effects, we performed high-throughput kinase activity mapping (HT-KAM),^26–28^ which enabled direct measurement of the catalytic activity of 165 kinases in avapritinib-treated high-grade glioma models (Figure 1A). We compared kinase activity in both acute and chronic treatment settings using complementary mouse and human systems. These included the PPK model, a syngeneic mouse-derived neurosphere culture generated via in utero electroporation (IUE) that incorporates a transposase and plasmids expressing dominant-negative *T**P**53*, D842V-mutant *P**DGFRA*, and *H3**K**27M*;^13^ and BT245, a *PDGFRA* H424Y/P422L and *H3K27M*-mutant cell line derived from an HGG patient. Parent PPK and BT245 cells were determined to have a 72-hour IC50 of 0.39 and 1.3 μM, respectively (Figure S1A). To model acquired resistance, cells were chronically exposed to escalating concentrations of avapritinib over several weeks until they achieved stable growth at 3 μM. This selection resulted in a ~2-fold increase in IC_50_ (to 0.75 μM) in one model and a ~3-fold increase (to 3.4 μM) in another (Figures 1B, S1A). For HT-KAM analyses, “short-term” conditions compared parental cells treated with 1 μM avapritinib for 8 hours to untreated controls, whereas “long-term” conditions compared chronically treated cells to their untreated parental counterparts.

The most prominent pathway that was upregulated was the MAP kinase (MAPK) pathway, which showed significantly higher kinase activity after both short and long-term avapritinib treatment (Figure 1C–E), despite being directly downstream of *PDGFRA* signaling (Figure S1B). Specifically, short-term avapritinib treatment for BT245 resulted in MEK/JNK (MAP2K4/MAP2K7) (log2FC = 4.1, p = 0.043) activation, and both short-term and long-term treatment resulted in sustained MEK2 (MAP2K2) (log2FC = 3.4, p = 0.003) activation in both models (Figure 1C–D). Multiple proteins that activate MAPK were also upregulated, including Aurora A kinase and the RTKs FGFR2 and PDGFRß, which demonstrated increased activity in response to both short- and long-term avapritinib exposure (Figure S1C). Of note, Aurora A kinase has been shown to indirectly activate the MAPK pathway through interactions with H-Ras.^29^

To further investigate the persistent MAPK signaling following avapritinib treatment, we leveraged “KPP” neurospheres, an additional IUE-syngeneic mouse-derived HGG model established to have high sensitivity to avapritinib^30^ (referred hereafter as “PPK*”). PPK* cells were chronically treated with avapritinib (0.15 μM; IC_80_). After one month, cells exhibited significantly higher IC_50_ upon re-exposure (Figure S1D). Bulk RNA sequencing of treated versus control cells, followed by pathway analysis using PROGENy data set, revealed selective enrichment of MAPK signaling relative to other pathways (Figure 1F), consistent with sustained MAPK activation despite PDGFRA inhibition. To determine if chronic avapritinib treatment resulted in genetic changes, we performed whole-exome sequencing (WES) of chronically treated PPK* cells. This demonstrated multiple high–variant allele frequency insertions in *PDGFRA*, each predicted to induce frameshifts and premature truncation of the receptor (Figure S1E), demonstrating selective pressure to disrupt PDGFRA signaling, but no *PDGFRA* variants seen in chronic avapritinib treatment of GI stromal tumors^23^ or in alternate RTKs/MAPK pathway genes. This is concordant with transcriptional profiling demonstrating robust MAPK pathway activation, supporting in this model that avapritinib response is mediated by pathway rewiring and engagement of MAPK-driven bypass signaling rather than restoration of PDGFRA activity or direct mutational activation of alternate RTKs.

**Figure 2:**
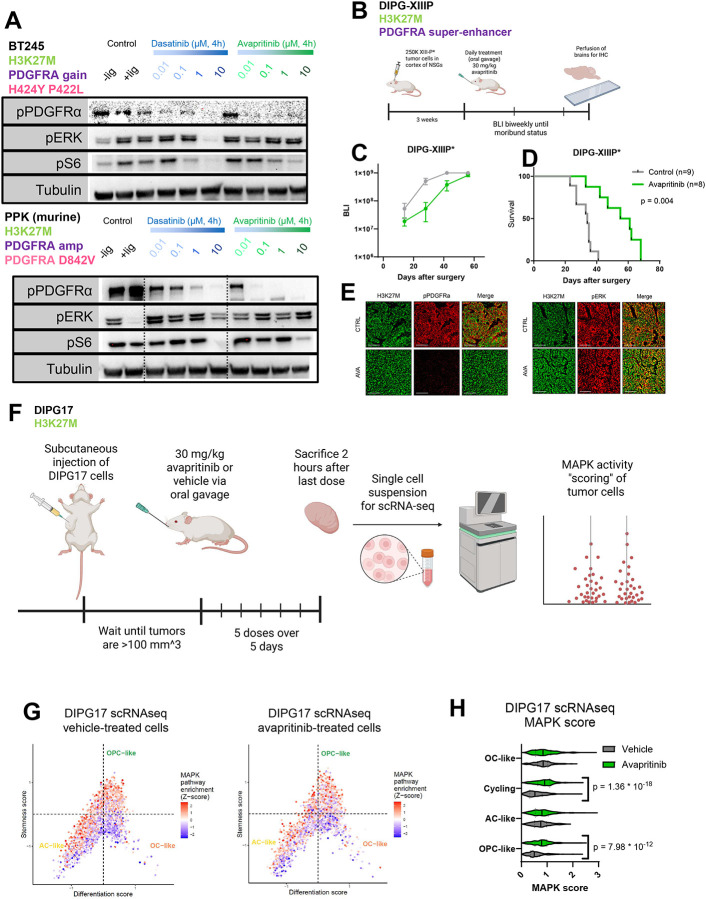
Identification of avapritinib-induced sustained MAPK activity in high-grade glioma models (**A**) Western blots of two pHGG cell models, examining phosphorylated PDGFRA and ERK expression after 4 hours of the TKIs dasatinib and avapritinib compared to control. Ligand-free control was deprived of growth factors overnight. (**B-D**) Schema, luminescence, and survival results of orthotopic cortex injections of DIPG-XIII-P* cells into NSG mice and treated with vehicle or avapritinib. (**E**) Multiplexed immunofluorescence of DIPG-XIII-P* tumors, showing H3K27M expression and phosphorylated PDGFRα (p-PDGFRα) expression following vehicle or avapritinib treatment. Scale bars indicate 100μM. (**F**) Schema of single-cell RNA-seq experimental setup of orthotopic implantation of DIPG17 cells in NSG mice. (**G**) Single-cell RNA-seq results of vehicle-treated and avapritinib-treated cells, mapped by differentiation and stemness. Color represents MAPK score. (**H**) MAPK scores of individual cell types from randomly subsampled scRNA-seq results.

Consistent with these results, though p-PDGFRα decreased with increasing inhibitor concentration, a persistence in p-ERK at inhibitor levels reaching complete PDGFRα inhibition was found by western blot with avapritinib treatment, but not with the PDGFRα inhibitor dasatinib (Figure 2A). Notably, dasatinib is less potent against PDGFRα than avapritinib (Figure S2A), and it is less selective for PDGFRα than avapritinib, with many more kinases inhibited.^8^ For further verification, the patient-derived SU-DIPG-XIII-P* cell model was selected for its high PDGFRα expression, which has been previously shown to be driven by “super-enhancer” activation.^31^ Orthotopic injections of DIPG-XIII-P* cells were performed in NSG mice; mice were allowed to recover for 2 weeks then underwent daily treatment of vehicle or avapritinib (Figure 2B). Avapritinib resulted in significantly lower luminescence at multiple timepoints and longer survival (Figure 2C–D), and multiplexed immunofluorescence (IF) revealed on-target p-PDGFRα inhibition (Figure 2E). Despite this, all mice succumbed to treatment, and tumors demonstrated sustained pERK activity despite treatment immediately prior to perfusion (Figure 2E).

On a cellular level, H3K27M-mutant diffuse midline glioma (H3K27M-DMG) harbor molecularly distinct subpopulations, with the oligodendrocyte precursor cell-like (OPC-like) cells representing the predominant cycling compartment and exhibiting the highest PDGFRα expression.^32,33^ To determine whether MAPK activation in response to avapritinib occurs preferentially within specific tumor cell populations, H3K27M-DMG SU-DIPG17 cells were injected in mice, and tumors were treated for 5 days with avapritinib or vehicle control prior to scRNA-seq^34^ (Figure 2F). In avapritinib-treated and vehicle-treated tumors, cells recapitulated the known cellular organization of DMG, with MAPK activity present across all subpopulations (Figure 2G). When comparing avapritinib treatment to control, MAPK activity was found to be significantly increased in cells overall (p = 1.24 * 10^−199^) (Figure S2B). When broken down by subtype, OPC-like (p= 7.98 * 10^−12^) and cycling (p= 1.36 * 10^−18^) tumor cells, but not the more differentiated oligodendrocyte-like (OC-like) or astrocyte-like (AC-like) populations, had higher MAPK activity (Figure 2H). Taken together, these results demonstrate that the MAPK pathway is activated in response to avapritinib most strongly in HGG cells with higher PDGFRα expression and innate sensitivity to avapritinib (OPC-like, cycling). These results support MAPK signaling as a context-dependent adaptive response to PDGFRα inhibition and a promising potential combinatorial target.

### Impact of PDGFRA variant type on avapritinib-induced MAPK activity

**Figure 3:**
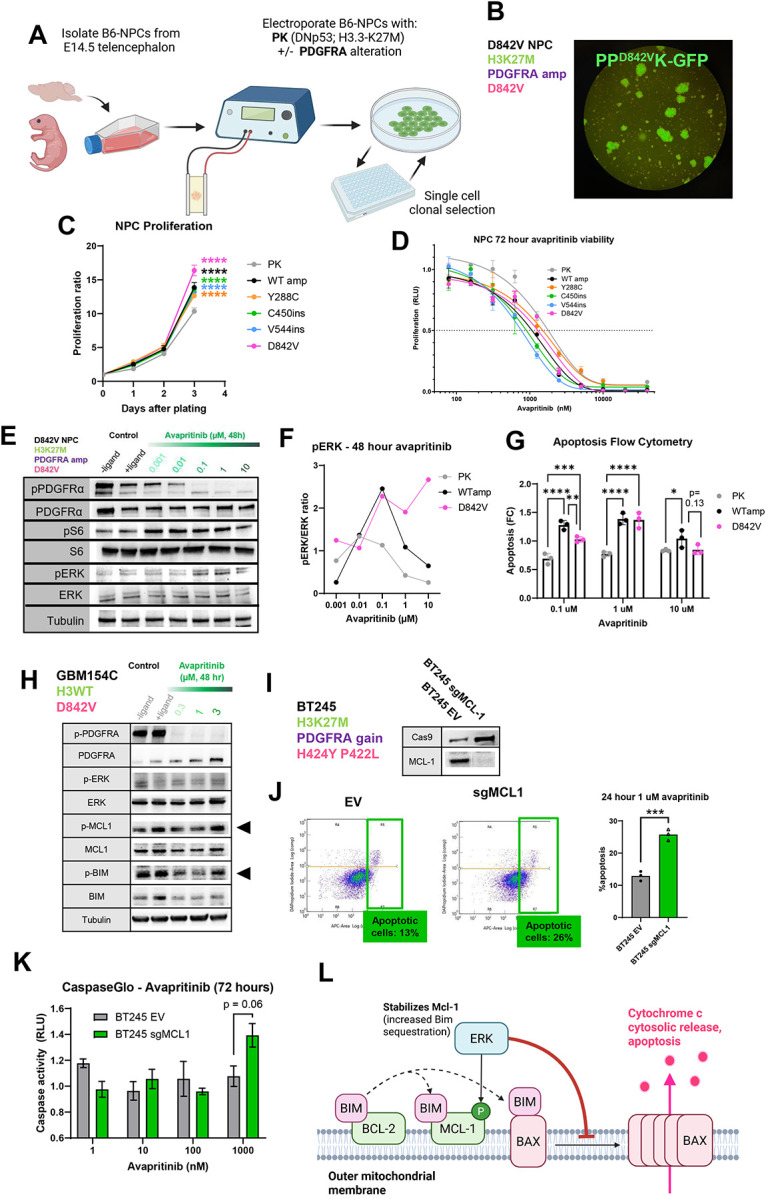
Impact of PDGFRA variant type on avapritinib-induced MAPK activity and MAPK impact on avapritinib-induced apoptosis (**A**) Schema of generation of neural progenitor cells (NPCs) expressing different variants of PDGFRA. (**B**) Image demonstrating positive GFP expression of NPC cells. (**C**) 72-hour proliferation curves of NPC variants. (**D**) 72-hour avapritinib dose response curve of NPC variants, and IC50 results. (**E**) 48-hour Western blot of PDGFRA D842V-mutant NPCs, examining phosphorylated PDGFRA, ERK, and S6 expression. (**F**) Quantification of pERK Western blots in (**E**), normalized to control. (**G**) Quantification of apoptosis as measured by Annexin V flow cytometry in D842V, wild-type, and PDGFRA-null (PK) NPCs. (**H**) Western blot of GBM154C cells after 4 hours of avapritinib treatment showing increase in ERK and downstream anti-apoptosis mediators. (**I**) Western blot results validating CRISPR KO of MCL-1 in BT245 cells. (**J**) Flow cytometry results of apoptosis as measured by Annexin V and DAPI stains at 24 hours of 1 μM avapritinib and quantification in graph form. (**K**) Apoptosis levels as measured by CaspaseGlo assay at 72 hours of 1 μM avapritinib. (**L**) Anti-apoptotic mechanism of ERK via stabilization of Mcl-1.

We recently reported that HGG is driven by a broad spectrum of *PDGFRA* alterations that can impact downstream signaling.^8^ Little is known about the impact of *PDGFRA* alteration type on avapritinib sensitivity or avapritinib-associated MAPK activation. To obtain isogenic control of clinically relevant PDGFRA variants, we generated novel *in vitro* models. Neural progenitor cells (NPCs) were harvested from embryonic mice and electroporated with plasmids harboring pHGG-driver genes (p53, H3K27M) as well as wild-type *PDGFRA* (amplified or “WT amp”) or recurrent *PDGFRA* mutations (Figure 3A–B). These models showed different proliferation rates, with all models showing higher proliferation than in control cells without *PDGFRA* alteration (“PK”) (Figure 3C, S3A). Further, these models showed greater sensitivity to avapritinib than PK controls, with D842V and Y288C showing the least sensitivity (Figure 3D, S3B), consistent with a previous publication reporting unique glycosylation and ER trapping of *PDGFRA* Y288C that result in drug resistance.^14^ These results suggest that PDGFRA mutational context influences response to targeted inhibition in DMG, although the mechanistic basis for the increased avapritinib sensitivity of PDGFRA^WT^ tumors remains unclear.

We further examined *PDGFRA* D842V, wild-type amplified, and control cells for differences in MAPK activation after avapritinib treatment. We observed a reduction in PI3K/mTOR activity (pS6) at 0.1 μM avapritinib across all models, whereas p-ERK levels were more variable in response to treatment (Figure 3E–F, S3C–D). Specifically, p-ERK increased between 0.01 and 0.1 μM for both WT and D842V models; this increase resolved at higher concentrations in the WT model yet continued to increase in the D842V model. We performed analysis of apoptosis via Annexin V flow cytometry in the setting of different concentrations of avapritinib for 24 hours; in both wild-type amplified and D842V mutant models, apoptosis levels were significantly higher at relevant concentrations of avapritinib than the PK controls, with apoptosis higher in WT than D842V at 0.1 μM and trending higher at 10 μM (Figure 3G). These data suggest that PDGFRA alterations exhibit differential MAPK activation with avapritinib therapy and that the D842V mutant may be particularly amenable to combination therapy targeting the MAPK pathway.

### MAPK impact on avapritinib-induced apoptosis

Next, we were interested in exploring the specific mechanisms of MAPK-induced changes in cellular response to avapritinib. In our previous work, we showed that 7 days of 1 μM avapritinib treatment leads to apoptotic cell death as assessed by Annexin V flow cytometry.^8^ Previous studies have demonstrated that MAPK pathway activity can promote anti-apoptotic signaling reducing cell death.^35^ In particular, ERK has been shown to phosphorylate both the anti-apoptotic protein MCL1, stabilizing it, and the pro-apoptotic protein BIM, targeting it for degradation; both of these effects lead to a decrease in overall apoptosis.^36–41^ We hypothesized that ERK-driven anti-apoptotic signaling may result in blunted cell death in response to avapritinib in HGG cells.

Indeed, *PDGFRA* D842V-mutant 154C human HGG cells showed increased phosphorylated MCL-1 and BIM between 1 and 3 μM after 48 hours of avapritinib treatment (Figure 3H). In cells grown in long-term sublethal concentrations of avapritinib, there was an increase in p-PDGFRα activity but a shift in phospho-ERK and a decrease in p-MCL1 activity (Figure S4A). To assess the role of MCL1 and BIM in sensitivity to avapritinib, we knocked out MCL1 and BIM in *PDGFRA*-altered BT245 cells (Figure 3I, S4B–C). BT245-sgMCL cells showed an increase in apoptosis at 1 μM avapritinib at 24 hours via flow cytometry (Annexin V) and a trend towards increased apoptosis at 1 μM avapritinib at 72 hours via Caspase 3/7 marker (Figure 3J–K, S4D–E). These data support that ERK-driven MCL1 activity can blunt apoptosis in response to avapritinib treatment in *PDGFRA*-driven HGG cells (Figure 3L).

### Combinatorial targeting of PDGFRA and MAPK pathways

We next tested whether targeting MAPK signaling could enhance the efficacy of avapritinib in HGG tumor cells. To do so, we selected three classes of MEK/ERK pathway inhibitors with established CNS penetration and prior use in brain tumor models: (1) imipridones ONC201 and ONC206, which have been shown to reduce ERK activity indirectly through the pro-apoptotic protein TRAIL^42^ and have shown recent promise in H3K27M-DMG tumors, with ONC201 FDA-approved for recurrent H3K27-altered DMGs;^43,44^ (2) MEK inhibitors trametinib and selumetinib, which have proven efficacy in BRAF-altered low-grade glioma;^45–47^ and (3) the ERK inhibitor ulixertinib, which was recently demonstrated to inhibit proliferation in glioma cells at low nanomolar concentrations.^48^

**Figure 4:**
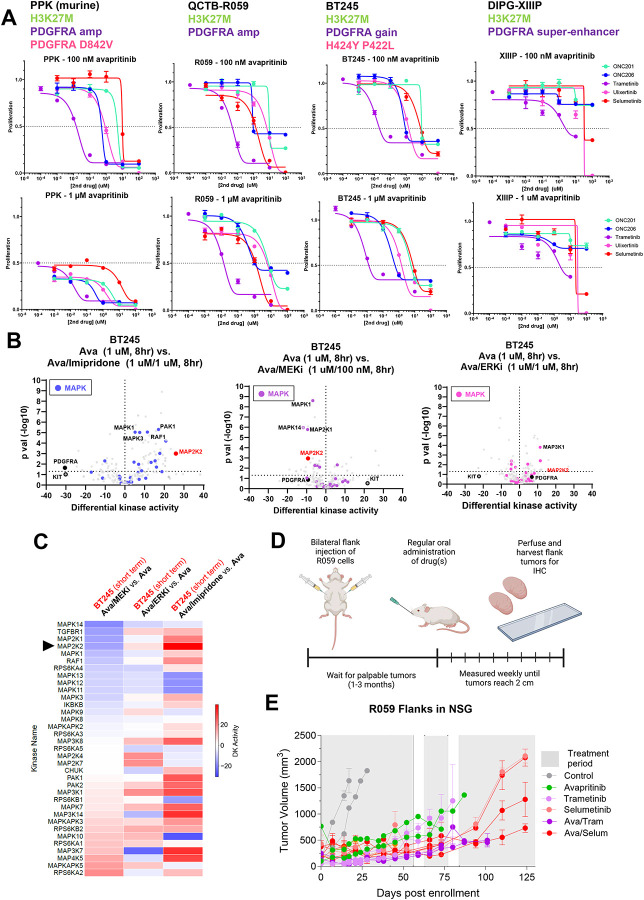
Combinatorial targeting of PDGFRα and MAPK pathways (**A**) 72-hour dose response curves using avapritinib and one of four MAPK-inhibiting drugs (ONC201, ONC206, trametinib, ulixertinib) in multiple pHGG cell models. 100 nM and 1 μM avapritinib dose response curves are selected for visualization here. (**B**) HT-KAM assay results comparing avapritinib monotherapy to combinatorial therapy (ONC201, trametinib, ulixertinib) in BT245 cells. (**C**) Heat map of HT-KAM results in (**B**). (**D**) Schema of experiment utilizing NSG mice injected in flank with R059 cells, treated with single or combinatorial therapies. (**E**) Flank tumor volume over time of mice by treatment group.

We previously treated mice with avapritinib at the human-equivalent dose of 60 mg/kg and observed peak plasma and brain concentrations of 20 μM.^8^ We therefore performed combinatorial therapy with avapritinib at 100 nM and 1 μM across PDGFRA-driven *in vitro* HGG models with various *PDGFRA* alterations (Figure 4A, S5A). Of note, the DIPG-XIII-P* cell model was found to be abnormally resistant across all drugs and combinations tested. For the other models, trametinib consistently resulted in IC50s between 0.01 and 0.1 μM when combined with avapritinib, ONC206 was between 0.1 and 1 μM, and ONC201 and selumetinib were between 1 and 10 μM. Although all drugs tested were effective at clinically relevant doses, the direct MEK inhibitor trametinib outperformed imipridones, ulixertinib, and selumetinib at multiple avapritinib doses *in vitro* (Figure 4A, S5A–B).

To assess impact on kinome signaling, we performed combinatorial HT-KAM analysis with short-term treatment using avapritinib and a second drug from each class of inhibitors: trametinib (MEK), ONC201 (imipridone) and ulixertinib (ERK). These experiments demonstrated a significant decrease in MEK activity with direct MEK inhibition but not with imipridone therapy or ERK inhibition (Figure 4B–C). Treatment of multiple *in vitro* models with avapritinib and either MEK inhibitor in the PPK model showed high synergism and effective knockdown of pERK activity with combination therapy (Figure S5C–D).

Based on this promising *in vitro* data, we implanted *PDGFRA*-amplified R059 cells in the flanks of NSG mice as a pilot experiment to assess tolerability and *in vivo* efficacy signal (Figure 4D). Control tumors took less than 30 days from being palpable to measuring greater than 2 cm in length, or moribund state. Mice treated with avapritinib, trametinib, and combination avapritinib/trametinib took between 75 and 100 days. Of note, avapritinib/trametinib-treated mice were euthanized prior to defined tumor size endpoints due to treatment-related toxicity and poor tolerance. Mice treated with selumetinib and combination avapritinib/selumetinib survived the longest, up to 125 days. Selumetinib and either MEKi combinatorial therapy with avapritinib were therefore the most effective treatment strategies in suppressing tumor growth (Figure 4E).

### Combinatorial therapy in orthotopic (brain) models of HGG

**Figure 5:**
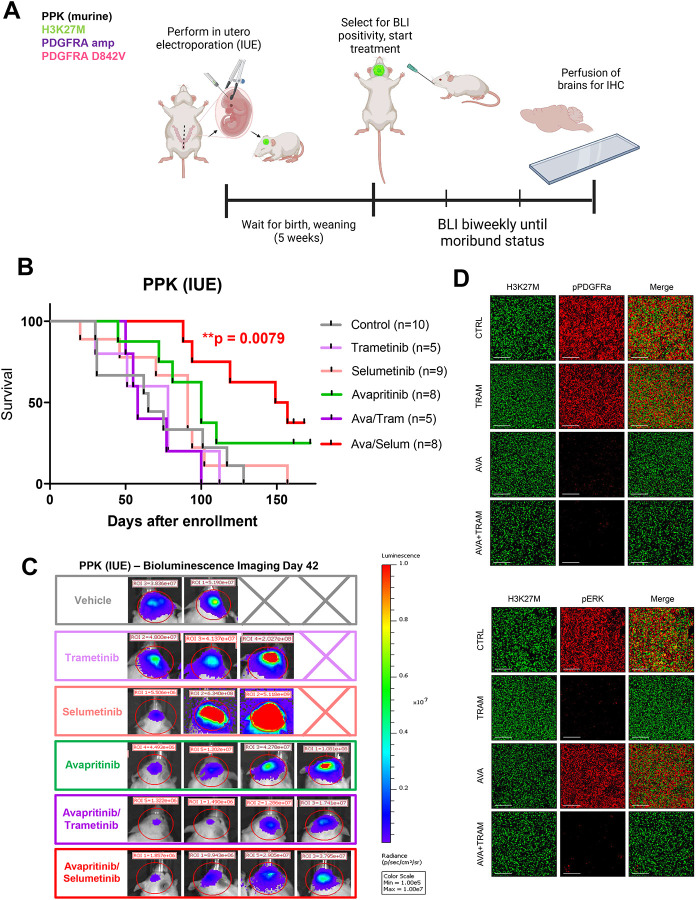
Combinatorial therapy in orthotopic (brain) models of HGG (**A-C**) Schema, survival, and luminescence results of in utero electroporation of CD1 mice with PPK plasmids, treated with vehicle, trametinib, selumetinib, avapritinib, avapritinib/trametinib, or avapritinib/selumetinib. (**D**) Multiplexed immunofluorescence analysis of brains from IUE mice perfused at moribund status 1 hour post-treatment.

Based on the promising *in vitro* and pilot *in vivo* data, we moved forward with *in vivo* experiments utilizing the combination of avapritinib and MEK inhibition. We generated PPK tumors (harboring the *PDGFRA* D842V mutation) via IUE (Figure 5A).^13^ Mice were treated with vehicle, avapritinib, avapritinib/trametinib, and avapritinib/selumetinib until moribund; bioluminescence of tumors was tracked biweekly. Results demonstrated that avapritinib/selumetinib-treated mice had a median survival of 149 days, significantly longer survival than control survival of 65 days (p = 0.0021); all other treatment groups did not have significantly longer median survival than control (Figure 5B). Avapritinib monotherapy and both combination therapies had lower luminescence values at days 28 and 42 of treatment (Figure 5C, S6A). Additional pilot treatment studies were done with orthotopic injection of PPK cells to assess on-target effect of avapritinib and MEK inhibitors or imipridones, which demonstrated downregulation of pERK activity in MEK inhibition and combination-treated mice compared to control, avapritinib, or imipridone monotherapy (Figure 5D, S6B–C). These data demonstrate that avapritinib and MEK inhibitor combination therapy is a viable, effective, and CNS-penetrant strategy for this tumor model, showing promise for this strategy in extending the survival of *PDGFRA*-altered HGG patients.

### Combinatorial avapritinib/MEK inhibitor therapy in human HGG patients

**Figure 6:**
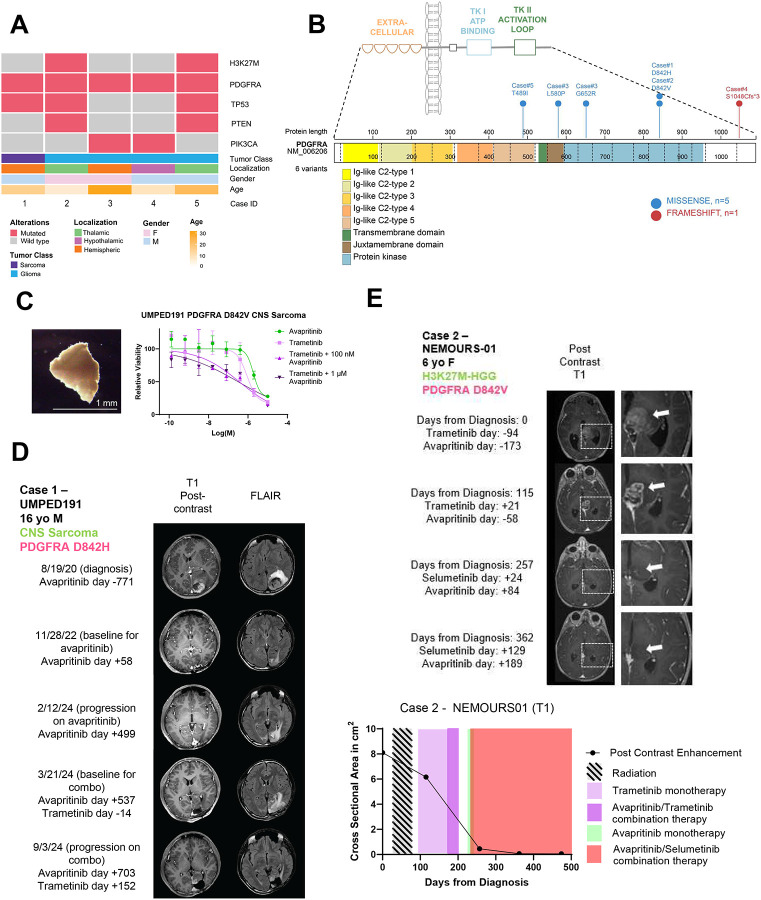
Combinatorial avapritinib/MEK inhibitor therapy in human HGG patients (**A**) Clinical summary of case patients treated with avapritinib/MEK inhibitor combination therapy. (**B**) Lollipop plot documenting *PDGFRA* mutations in clinical cases along PDGFRα protein structure. (**C-D**) Case 1 (UMPED191), a 16-year-old male with H3WT PDGFRA-D842H CNS sarcoma treated with avapritinib monotherapy initially before switching to avapritinib/trametinib co-therapy. (**C**) Tumor organoid, screening dose response curve, and (**D**) representative T1 and FLAIR MRI images are shown. (**E**) Case 2 (NEMOURS01), a 5-year-old female with H3K27M PDGFRA-D842V DMG treated briefly with avapritinib and trametinib and subsequently maintained on avapritinib and selumetinib. Representative T1 MRI images and enhancing tumor area over time are shown.

Motivated by the efficacy observed in preclinical models, we evaluated combined PDGFRA and MAPK pathway inhibition in patients with PDGFRA-altered brain tumors who had progressed on standard-of-care therapy and lacked suitable clinical trial options. Avapritinib was administered in combination with a MEK inhibitor (trametinib or selumetinib, at the clinician’s choice), with drugs obtained through commercial supply. Clinical data were collected from five patients treated across four institutions under an IRB-approved protocol at the University of Michigan, including avapritinib plus trametinib (n=5) and avapritinib plus selumetinib (n=1), with one patient receiving both regimens (Figure 6A–B; Table 1). Dosing was per weight equivalent dosing of adult therapy for GIST and established regimens for MEK inhibitors in low-grade glioma. In terms of tolerability, combination therapy of avapritinib plus trametinib was associated with increased adverse events, including edema, cytopenias, and gastrointestinal symptoms, necessitating dose reduction or treatment interruption in 2 of 4 patients (Table 2).

Cases 1 and 2 were prioritized for in-depth analysis to functionally interrogate PDGFRA-targeted combination therapy. Case 1 (UMPED191), a 16-year-old male with multiply recurrent PDGFRA D842H-mutant CNS sarcoma, experienced disease progression following multimodal therapy, including radiation, cytotoxic chemotherapy, and re-irradiation. Avapritinib monotherapy achieved a transient period of disease stability (16 months), after which progression prompted tumor resection and establishment of a patient-derived organoid (PDO). *Ex vivo* profiling demonstrated relative resistance to avapritinib at nanomolar concentrations but marked sensitization with MEK inhibition, even at low-dose trametinib (Figure 6C). Clinically, dual avapritinib/trametinib recapitulated this combinatorial effect, resulting in short-lived disease stabilization; however, treatment was limited by toxicity and ultimately followed by progression (Figure 6D, S7A).

Case 2, a 6-year-old female with H3K27M-mutant, *PDGFRA* D842V diffuse midline glioma, further supports the context-dependent benefit of MAPK co-targeting. Following radiotherapy and a brief course of trametinib, combination avapritinib/trametinib was discontinued due to grade 4 dermatologic toxicity, which was deemed likely related to trametinib. Substitution with selumetinib enabled sustained dual pathway inhibition, yielding a 40% tumor reduction and prolonged disease control over 9 months (Figure 6E, S7B). Notably, progression-free survival of 17 months from diagnosis exceeds historical benchmarks for H3K27M diffuse midline glioma (7–10 months),^49,50^ suggesting potential clinical activity of PDGFRA and MAPK co-inhibition in this molecular context.

Within the limits of this small case series, these cases support that PDGFRA-mutant pediatric brain tumors may exhibit incomplete dependency on PDGFRA signaling alone, with enhanced therapeutic vulnerability revealed through concurrent MAPK pathway inhibition.

## Discussion

Avapritinib has been shown to have initial promise as a therapeutic strategy for *PDGFRA*-altered HGG; however, pre-clinical tumors and case series of patients have shown that all tumors eventually progress.^8^ To optimize combinatorial therapy, we performed kinome activity analysis to identify the MAPK pathway as a downstream kinase-containing pathway being activated by avapritinib therapy. This pathway has been implicated in TKI resistance in other cancers and linked to anti-apoptotic activity downstream of ERK.^35^ Here, we demonstrate mechanistic advancement of crosstalk signaling leading to anti-apoptotic blunting of avapritinib response and the combinatorial benefit of MAPK-targeting therapies.

When comparing different *PDGFRA* variants, our work and others have shown that the alteration type can strongly impact tumor signaling and TKI therapy response.^8,12^ In Bahlawane et al., p-ERK activation in response to the TKI imatinib was found to be enhanced in D842V compared with D842Y or V561D in GIST cells, along with decreased imatinib sensitivity in D842V.^51^ Avapritinib has previously been found to effectively target the kinase domain mutation D842V, a target that eluded other inhibitors in class; however, the drug has activity across a wide spectrum of *PDGFRA* variants.^22^ Because avapritinib is a type I kinase inhibitor and inhibits the active conformation, it may be inherently primed to inhibit overactive PDGFRα in any tumor with increased PDGFRα signaling, regardless of specific variant. Nevertheless, some mutations have proven more resistant to drug therapy. The PDGFRα external domain mutation (Y288C) was shown to result in ligand-independent dimerization in intracellular organelles and reduced degradation, leading to resistance;^14^ this was also described with the V561D and D842V mutations.^51^ The ATP-binding mutation N659K was found to confer a 10-fold increase in IC50 to avapritinib.^23^ These mutations may not be targetable by avapritinib alone, but work has been done to identify additional pockets potentially targetable by next-generation inhibitors.^52^ Furthermore, our work demonstrating dose-dependent increased MAPK signaling in avapritinib-treated D842V models suggests that combinatorial therapy may have particular benefit in this variant. Our data on variant targetability will inform what inhibitors to use for patients with specific *PDGFRA* variants and which combinatorial therapeutic strategy may be warranted.

The Raf-MEK-ERK pathway regulates multiple downstream pathways for cell growth and differentiation; identifying the most relevant downstream effect is difficult.^53^ In other solid tumors, resistance to EGFR-targeting TKIs has been associated with ERK-mediated stabilization of the downstream anti-apoptotic protein MCL-1 and destabilization of the pro-apoptotic protein BIM.^35^ We found that long-term avapritinib exposure increases both ERK and MCL-1 activity, and that knocking out MCL-1 increases susceptibility of HGG models to apoptosis. Our work opens the door to future studies to explore the effect of tumor type or PDGFRA variant type on ERK or MCL1 and other pro- and anti-apoptotic proteins (BCL-2, BIM, etc.) in HGG models, as well as ERK’s many transcription factor targets (e.g. c-Myc, Ets-1, c-Jun).^53^

While our clinical case series is limited and heterogeneous, there are a few important potential insights. Case 1 was stable on avapritinib monotherapy for almost 2 years before progressing. Collecting tissue and testing drug sensitivities *in vitro* then identified a potential benefit from therapeutic combination with a MEK inhibitor, which he remained stable on for 4 months. It is unclear whether this combinatorial strategy ultimately had any effect, but this case nonetheless demonstrates the need for optimizing combinatorial therapies in this subset of patients who are already primed to be treatment-refractory. Case 2 demonstrates the potential utility of optimally treating patients with an avapritinib-based combinatorial regimen after initial radiation rather than waiting for progression, as was seen in Case 1 of our prior monotherapy cohort who had a complete response of primary tumor to avapritinib.^8^ As well, Case 2 demonstrates the potential benefit to tolerability of the avapritinib-selumetinib combination compared to avapritinib-trametinib. Selumetinib binds to a unique allosteric site on MEK1 and MEK2, conferring a relatively high selectivity.^54^ Trametinib, being more potent than selumetinib, also promotes dissociation of MEK1/2 from c-Raf, reducing rebound of ERK phosphorylation and providing more complete MAPK knockdown.^55^ This complete knockdown may lead to stronger MAPK inhibition in non-cancerous cells, which would result in off-target effects in patients, particularly dermatological effects, and require cessation of treatment.^56,57^ These factors may make selumetinib the more favorable choice when it comes to CNS-penetrant MEK inhibitors in combination with avapritinib.

Of note, human pharmacologic studies with avapritinib show a mean steady state C_max_ of avapritinib of 813 ng/mL (2.0 μM) following administration of avapritinib 300 mg once daily.^58^ Avapritinib is highly (98.8%) protein-bound, and its binding is concentration-independent.^59^ Our previous *in vivo* data demonstrated higher brain than plasma penetration.^8^ Future studies on the non-protein-bound brain tumor concentrations of avapritinib will help clarify optimal dosing to model chronic treatment. Nevertheless, our robust combinatorial *in vivo* data demonstrate the significant promise of dual avapritinib-MEK inhibitor treatment.

In summary, avapritinib and MAPK inhibition combination therapy is a viable potential therapeutic strategy for high-grade glioma patients with *PDGFRA* alterations rather than targeted monotherapy with avapritinib. As severely limited as treatment options are for this patient population, targeted therapy remains a hopeful avenue for improving survival for this deadly disease.

## Methods

### High-throughput kinase activity-mapping (HT-KAM)

The high-throughput kinase-activity mapping (HT-KAM) platform uses arrays of peptides that act as combinatorial sensors of the phosphorylation activity of kinases. The phospho-catalytic signature of samples is established from simultaneously occurring ATP-consumption tests measured in the presence of individual peptides that are experimentally isolated from each other. Assays were run in 384 well-plates, where each experimental well contains one peptide. The final 8μL reaction mixtures per well contain: (a) kinase assay buffer (1X KaB: 2.5mM Tris-HCl (pH7.5), 1mM MgCl_2_, 0.01mM Na_3_VO_4_, 0.5mM β-glycerophosphate, 0.2mM dithiothreitol (DTT), prepared daily; (10X KaB Cell Signaling Technology^®^, Cat.No. 9802), (b) 250nM ATP (prepared daily with 1X KaB; Cell Signaling Technology^®^ Cat.No. 9804), (c) 200 μg/ml 11-mer peptide (lyophilized stocks originally prepared as 1 mg/ml in 1X KaB, 5% DMSO), and (d) samples made from cell at −10 μg/ml total protein extract. Samples are kept on ice and diluted in 1X KaB < 30 min before being used. Controls with no-ATP, or no-peptide, or no-sample as well as ATP standards are run side-by-side within each 384 well-plate. High-throughput liquid dispensing of all reagents is performed using a Biomek^®^ FX Laboratory Automation Workstation from Beckman Coulter. All reagents are kept on ice and plates on cold blocks until enzymatic reactions are started. Once the dispensing of the reaction mixtures is complete, the plates are incubated for 1 h at 30°C. ATP is detected using Kinase-Glo revealing reagent (Promega^®^; Cat.No V3772), which stops the activity of the kinases and produces a luminescent signal that directly correlates with the amount of remaining ATP in the samples. Luminescence is acquired using the Synergy 2 Multi-Mode Microplate Reader from BioTek. Luminescence data are inversely correlated with the amount of kinase activity. The activity of kinase enzymes is derived from their respective subset of biological peptide targets included in the assay.

### Cell lines

Mouse HGG primary “PPK” cells ([I] PBase, [II] PB-CAG-DNp53-Ires-Luciferase (dominant negative TP53 or TP53 hereafter), [III] PB-CAG-PdgfraD824V-Ires-eGFP (*PDGFRA* D842V), and [IV] PB-CAG-H3.3 K27M-Ires-eGFP (H3K27M)) were generated by harvesting intra-uterine electroporation (IUE) tumors at the time of euthanasia, using established methodology.^13,60^ Tumors were located by green fluorescent protein (GFP) expression under an epi-fluorescent microscope (Olympus CKX41) at the time of resection. The tumor mass was gently homogenized and dissociated with non-enzymatic cell dissociation buffer (Gibco). Cell suspension was filtered through a 70 μm cell strainer, centrifuged at 300 × g for 4 min, and resuspended into 7 ml of Neurobasal-A Medium (1x) Base media (Invitrogen, 500 mL). Each base stock of media was supplemented with 5 mL B27 without vitamin A, 5 mL Antibiotic-Antimycotic, 500μL Heparin, 500 μL EGF (20ng/mL), 500 μL FGF (20ng/mL), 250 μL of PDGF-AA and PDGF-BB (10 ng/mL).

KPP (H3.3^K27M^, p53^LOF^, PDGFRA^WT^) cells, referred to as PPK* in text, were obtained ex-vivo from IUE mouse model as previously described,^30^ grown at 37°C, 5% CO2 into NeuroCult NSC proliferation media (STEMCELL Technologies) containing 20 ng/ml of recombinant-epidermal growth factor (Miltenyi Biotec) and recombinant-fibroblast growth factor (Miltenyi Biotec), 10 ng/ml recombinant Human-PDGF-AA (Shenandoah Biotechnology), 10 ng/ml recombinant Human-PDGF-BB (Shenandoah Biotechnology) and 2 μg/ml Heparin Solution (STEMCELL Technologies) and were grown in 3-D as neurospheres using ultra-low attachment plates (Corning).

KPP resistant cells were established by exposing the parental cells to a constant concentration of Avapritinib of 0.15 μmol/L (IC_80_ value). Cells for the control were maintained in DMSO for only two passages to keep the baseline clonal population as close as possible to the parental culture. Of note, KPP cells were determined by whole exome sequencing to have obtained an endogenous D842V mutation not induced by IUE.

Patient derived glioma lines (BT245, R059, DIPG-XIII-P*, 154C) were grown as neurospheres in tumor stem media as described previously.^61^ IUE-generated *PDGFRA*-driven HGG primary cell culture (PPK) were cultured in conditions as described above. Cell lines were tested for mycoplasma every two months. QCTB-R059 and DIPG-XIII-P* cells were provided by Dr. Michelle Monje (Stanford); QCTB-R059 were initially generated by Dr Andy Moore’s lab (University of Queensland), Mayo 154C was derived from PDX models kindly provided by Dr. Jann Sarkaria.^13,62^

### Immunoblots

Treated cells were lysed in RIPA buffer containing phosphatase and protease inhibitors and immunoblotting was performed. Membranes were probed with primary antibodies overnight: PDGFRα, pPDGFRα (Y754), S6, pS6 (S235/236), AKT, pAKT (S473), ERK, pERK (T202/Y204), MCL1, pMCL1 (S159/T163), tubulin; PDGFRα and pPDGFRα required several nights of primary incubation for some cell models. Secondary antibodies were incubated for 1 hour and detected with iBright CL1000 (Invitrogen). Immunoblot results were quantified with area measurements using ImageJ.

### Resistance assays

KPP cells were plated in 96-well plates and treated with Avapritinib at different concentrations for 6 days. At the endpoint cell viability was measured using CellTiter-Glo (2.0 Promega). Relative luminescence units (RLU) for each well were normalized to the median RLU from the DMSO control wells as 100% viability. Three technical replicates per condition were performed. IC50 values (drug concentration causing 50% inhibition of cell proliferation) were calculated using GraphPad Prism, and the curves show the mean ± SD of the replicates per condition measured. Avapritinib was purchased from MedChemExpress (MCE) and were diluted in DMSO to a final concentration of 10 mmol/L.

### Nucleic Acid Extraction

DNA was extracted from cell pellet using Wizard Genomic DNA purification kit (Promega). RNA was extracted using the RNeasy Mini Kit protocol (Qiagen, 74104). DNA and RNA concentration and quality were measured using a Nanodrop spectrophotometer (Thermo-Scientific). RNA integrity and quality was analyzed using Qubit 4 (software version 2.18).

### Whole-Exome Sequencing

Whole-Exome sequencing was performed in collaboration with the CRUK Cambridge Institute Genomics core. Briefly, exome libraries were prepared from 400 ng genomic DNA using the Illumina DNA Prep with Exome 2.5 v04 with Twist Mouse Exome, following the manufacturer’s instructions. Libraries were pooled prior to enrichment and amplified with 9 PCR cycles pre-capture and 12 cycles post-capture. Captured libraries were quantified and quality controlled prior to sequencing. Paired-end sequencing (2 × 150 bp) was performed on an Illumina NovaSeq X using a 1.5B flow cell. The sequencing analysis was performed in collaboration with the CRUK Cambridge Institute Bioinformatics core. Briefly, Raw FASTQ files were quality-assessed using FastQC v0.12.1 (Andrews, 2010). Adapter sequences and low-quality bases were removed using fastp v0.23.4.^63^ Trimmed reads were aligned to the GRCm38 reference genome using the with bwa-mem2 v2.2.1.^64^ Duplicate reads were marked using Picard v3.1.1 (Broad Institute; https://broadinstitute.github.io/picard/). Somatic single nucleotide variants (SNVs) and small indels were called using the nf-core/sarek pipeline v3.4.0.^65^ Variant calling was performed with GATK Mutect2 in tumour only mode (bundled within nf-core/sarek).^66^ Filtered SNVs from Mutect2 were subject to additional quality filtering using the using GATK VariantFiltration applying the following criteria (variants failing any filter were excluded from downstream analysis):Variant allele count in tumour < 3 (VariantAlleleCount < 3), Variant allele count in matched normal > 1 (VariantAlleleCountControl > 1), Median mapping quality of variant-supporting reads < 40 (VariantMapQualMedian < 40.0), Median mapping quality difference (variant vs. reference reads) outside −5 to +5 (MapQualDiffMedian < −5.0 || > 5.0), Proportion of low-mapping-quality reads > 5% (LowMapQual > 0.05), Median base quality of variant-supporting reads < 25 (VariantBaseQualMedian < 25.0). Variants were annotated with the Ensembl Variant Effect Predictor (VEP) v102^67^ using the GRCm38 release 102 cache. To identify candidate drug-resistance mutations, variants detected in treated samples were filtered to remove any variant also found in the matched DMSO vehicle control sample from the same cell line. Position-based filtering: variants at any genomic position (chromosome and coordinate) present in the matched control were excluded from the treated sample. Gene-based filtering: variants in any gene with any variant detected in the matched control were excluded from the treated sample. Avapritinib treated samples were matched to their respective DMSO control. Per-transcript and highest-impact-per-position summary outputs were produced. Filtering was applied independently to variants called by the standard per-sample Mutect2 pipeline and the joint calling pipeline.

### RNA-seq

RNA-sequencing (RNA-Seq) was performed in collaboration with the CRUK Cambridge Institute Genomics core. Briefly, total RNA was isolated from cells using RNeasy Plus Mini Kit (Qiagen), and 500 ng of purified RNA were sent for analysis. RNA integrity and quality was analyzed using Qubit 4 (software version 2.18) following the manufacturer's recommended protocol. RNA-seq libraries were prepared from 249–600 ng total RNA using the Illumina Stranded mRNA Prep v04 kit according to the manufacturer’s instructions. Libraries were amplified with 11–12 PCR cycles, quantified, and quality controlled prior to sequencing. Paired-end sequencing (2 × 50 bp) was performed on an Illumina NovaSeq X using a 1.5B flow cell. The sequencing analysis was performed in collaboration with the CRUK Cambridge Institute Bioinformatics core. Briefly, raw FASTQ files were assessed for read quality using FastQC v0.12.1 (Andrews S. *FastQC: a quality control tool for high throughput sequence data*. Babraham Bioinformatics, Babraham Institute, Cambridge, UK. https://www.bioinformatics.babraham.ac.uk/projects/fastqc/). Transcript abundance was quantified using Salmon v1.10.3^68^ against the GENCODE M31 transcriptome (GRCm39).^69^ Differential gene expression analysis was performed using DESeq2 v1.42.0 (Bioconductor 3.18).^70^ Signalling pathway activity was estimated from DESeq2 Wald test statistics using a multivariate linear model implemented with decoupleR v2.8.0.^71^ The PROGENy (Pathway RespOnsive GENes) prior knowledge gene set resource was used.^72^

### DIPG17 flank model treatment study and scRNA-seq analysis

Juvenile NOD.Cg Prkdcscid/J (NSG^™^, Envigo) mice were inoculated with 500,000 DIPG17-luciferase tagged cells in 1:1 suspension (200 μl total) with full media and Matrigel^™^ (Fisher Scientific CB-40234) via subcutaneous injection in the left flanks, as previously published.^34^ Two weeks post-injection, animals were injected intraperitoneally with luciferin and scanned with PerkinElmer IVIS Optical Imaging to monitor tumor growth. Animals were monitored biweekly until tumor size reached ~100 mm^3^ (range of volumes 75 – 185 mm^3^) and tumor-bearing mice were randomly assigned to distinct drug arms or vehicle control treated groups when they reached adequate size. Animals were treated for 5 days at the following doses: Vehicle (n=4) (N-Methyl-2-pyrrolidone (NMP) as solvent and 30%PEG-400 + 5% Tween 80 + 65% D5W (PTD) or avapritinib (n=2) 30mg/kg PO QD. Mice were euthanized for tumor isolation and scRNA-seq 2 hours after the last dose was given at day 5.

After tumor isolation, fat, fibrous and necrotic areas were removed from the tumor sample in a tissue culture hood. Then, tumor was cut into 1–2 mm pieces and transferred to a gentleMACS C Tube (Miltenyi Biotec #130-093-237) containing 4.7 mL of sterile media (Knockout DMEM/F12), 200 μl of Enzyme H, 100 μl of Enzyme R, and 25 μl of Enzyme A from the human Tumor Dissociation Kit (Miltenyi Biotec #130-095-929). C Tubes were placed in the gentleMACS Dissociator under the “Medium” Tumor type (gentleMACS Program 37C_h_TDK_2) for 18 minutes. Then, samples were filtered through a 70μm strainer (previously primed with 2 ml of sterile media). Samples were centrifuged at 250 *x g* at 4°C for 8 minutes. Then, resuspended in 400 μl of red blood cell (RBC) lysis buffer (Miltenyi Biotec #130-094-183) and incubated on ice. After 2 minutes, at least 3 times the volume of RBC was added to rebalance osmolarity and the sample was centrifuged at 250 *x g* at 4°C for 5 minutes and resuspended in HBBS + 0.04% BSA. Samples were then examined for cell viability and counts using the Countess II. Samples were then brought to the Columbia University Single Cell Analysis Core where libraries were prepared following the manufacturer’s user guide and sequenced on the Illumina NovaSeq 6000 Sequencing System. ScRNA-seq data were processed with Cell Ranger software’s default parameters. Cell Ranger performed default filtering for quality control, and produced a barcodes.tsv, genes.tsv, and matrix.mts file containing transcript counts for each sample. These data were loaded into the R version 4.2.2. for further analyses. For MAPK score analysis, gene set enrichment analysis was performed using a publicly available list of MAPK genes from KEGG. Subpopulations were randomly sampled to include 240 cells per subpopulation. All p-values were calculated doing Wilcoxon test, and adjusted with Bonferroni.

### Dose response curves and Caspase-Glo assay

Cells were plated in 384-well plates (Corning) at 500–1000 cells in 25 ul of media (tumor stem medium as described previously) on day 0, depending on cell line growth rate. Drug was dissolved and diluted per desired concentration in 25 ul of media on day 1. On day 4 (72 hours after treatment), 50 ul CellTiter-Glo (Promega) or Caspase-Glo 3/7 (Promega) was added, and luminescence of plates were read using the Synergy HTX Multi-Mode Microplate Reader (BioTek Instruments).

### PDGFRA variant NPC generation

Pregnant C57BL6/J mice were ordered from Charles River Laboratories. At day E14.5, embryos were removed from the uterus, and brains were manually dissected and plated in flasks with tumor stem medium. NPCs were allowed to grow for 5 days and then electroporated with plasmids used in IUE (see IUE section of methods) using Lonza Nucleofector 2b per manufacturer protocol. Of note, the *Pdgfra-D842V* plasmid was modified to code instead for different *PDGFRA* variants or was not included in the electroporation for control cell line (PK). Cells were diluted to single-cell clonal selection in 96-well plates, and clones with strong GFP expression were selected and grown up into culture.

### Cell Line Nanopore WGS and copy number analysis

For confirmation of *PDGFRA* copy number, genomic DNA was extracted from R059 cells using Qiagen DNeasy Blood and Tissue DNA extraction kit (#69504) according to manufacturer's instructions. Resulting DNA (800–1000ng) was sequenced using Oxford Nanopore Technologies (ONT) MinION sequencer (#SQK-NBD114.24) according to manufacturer instructions. Resulting sequencer output was basecalled using ONT Guppy basecaller (v6.5.7) and aligned to the human reference genome (hg19) using minimap2 (v2.17). To estimate *PDGFRA* copy number increases in R059 cells, we used BioRad QX200 droplet-digital PCR (ddPCR) system and a BioRad *PDGFRA* copy number estimation assay (#10031240, #10031243). The assay was performed in triplicate and resulting copy numbers were determined in the QuantaSoft software (v1.7.4) by isolating distinct populations of droplets. To estimate copy number, we applied the CoRAL long read copy number analysis toolkit to the aligned WGS data using default parameters. Code is available at: AmpliconSuite/CoRAL: https://github.com/AmpliconSuite/CoRAL

### Apoptosis Assay

Cell lines were treated with 1 and 10 μM avapritinib and analyzed 24 hours after drugging. For apoptosis analysis, cells were stained with AnnV APC and Dapi (Thermofisher). FACS measurements were performed on BioRad ZE5 Cell Analyzer and analyzed with FlowJo.

### R059 flank study

Juvenile NSG mice (6–8 weeks) were inoculated with 500,000 QCTB-R059-GFP tagged cells in 1:1 suspension (200 μl total) with full media and Matrigel^™^ (Fisher Scientific CB-40234) via subcutaneous injection in both flanks. Animals were monitored biweekly until tumor size reached ~100 mm^3^, and tumor-bearing mice were randomly assigned to distinct drug arms or vehicle control treated groups when they reached adequate size. Mice were treated with: (a) vehicle (5% N-methyl pyrrolidone, 55% D5 water, 30% PEG-300, 10% Tween-80) PO, (b) 30 mg/kg avapritinib PO daily 5x/week, (c) 50 mg/kg ONC206 PO 2x/day 3x/week, (d) 1 mg/kg trametinib PO daily 5x/week, (e) 75 mg/kg ulixertinib PO 2x/day 5x/week, (f) avapritinib and ONC206, (g) avapritinib and trametinib, or (h) avapritinib and ulixertinib (previous doses, routes, and schedules used). Tumors were measured weekly until surpassing 2 cm in any dimension, and mice were euthanized at this stage.

### DIPG-XIII-P* orthotopic study

2.5×10^5^ cells DIPG-XIII-P* cells were injected stereotactically into the cortex of 6-week old female NSG mice. The skull of the mouse was exposed through a small skin incision, and a small burr hole was made using a 25-gauge needle at the selected stereotactic coordinates zeroed on lambda: −2.0 mm X, +1.0 mm Y and 2.5 mm Z. Cells were loaded into a 33-gauge Hamilton syringe, and injected at a rate of 0.5 μl/min with use of an infusion pump. Upon completing injection, the needle was left in place for another minute, then withdrawn slowly to help reduce cell reflux. After closing the scalp with suture and staple, mice were returned to their cages placed on a warming pad and visually monitored until full recovery. Tumor growth was monitored by bioluminescence (BLI).

### PPK orthotopic study

2.5×10^5^ cells PPK (IUE-generated) cells were injected stereotactically into the brainstem of 6-week old female C57BL6/J mice. The skull of the mouse was exposed through a small skin incision, and a small burr hole was made using a 25-gauge needle at the selected stereotactic coordinates zeroed on lambda: 0.8 mm X, −1.0 mm Y and 2.5 mm Z. Cells were loaded into a 33-gauge Hamilton syringe, and injected at a rate of 0.5 μl/min with use of an infusion pump. Upon completing injection, the needle was left in place for another minute, then withdrawn slowly to help reduce cell reflux. After closing the scalp with suture and staple, mice were returned to their cages placed on a warming pad and visually monitored until full recovery. Tumor growth was monitored by bioluminescence (BLI).

### Murine model of HGG using IUE

IUE was performed using sterile technique on isoflurane/oxygen-anesthetized pregnant CD1 female mice at E13.5 (cortex), according to established methodology.^13^. All tumors for this study were generated by injecting plasmids into either the lateral ventricle (forebrain) or the fourth ventricle (hindbrain). In this study, we injected the following 4 plasmids together: (a) PBase, (b) PB-CAG-DNp53-Ires-luciferase (dominant-negative TP53, referred to herein as just TP53), (c) PB-CAG-*Pdgfra*D824V-Ires-EGFP (*PDGFRA* D842V), and (d) PB-CAG-H3.3 K27M-Ires-EGFP (H3K27M), referred to herein as the PPK model.

Following anesthesia induction, carprofen was administered subcutaneously for additional analgesia. Uterine horns were exposed through a 1 cm incision, and individual embryos were digitally manipulated into the correct orientation for intraventricular injection. A pulled capillary needle was loaded with endotoxin-free DNA and Fast Green Dye (0.05%, MilliporeSigma) for visualization, and a microinjector was used to inject either the lateral or fourth ventricles with the DNA-dye mixture. 3 to 4 plasmids were injected simultaneously, each at a final concentration of 1 μg/μL, and 1–2 μL total solution was injected per embryo. DNA was then electroporated into cortical neural progenitors using 5 mm tweezertrodes (BTX), applying 5 square pulses at 35 V, 50 ms each at 950 ms intervals. The embryos were then returned into the abdominal cavity, the muscle and skin were sutured, and the animal was monitored until fully recovered from the procedure.

After delivery, the efficacy of plasmids uptake was monitored by bioluminescence using the IVIS Spectrum (Caliper Life Sciences) (tumors express luciferase). Pups that did not display bioluminescence (approximately 5%–10% of the pups) were euthanized. After 3 weeks, the juveniles were weaned and separated by sex. The mice with a positive signal were monitored for tumors every day by observation and biweekly by bioluminescence imaging on an IVIS Spectrum imaging system until the signs of intracranial tumor burden ensued.

### In vivo treatment – DIPG-XIII-P*, PPK, and IUE studies

Mice were treated when tumors reached a logarithmic growth phase (minimum 2 × 10^5^ photons/second via bioluminescence imaging or flank tumors measuring >100 mm^3, and confirmation was made that the treatment groups had equivalent average luminescence at the time of treatment.

Mice litters from each experimental group were randomized to (a) vehicle treatment (5% N-methyl pyrrolidone, 55% D5 water, 30% PEG-300, 10% Tween-80) PO, (b) treatment with 30 mg/kg avapritinib PO, (c) 1 mg/kg trametinib PO, (d) 25 mg/kg selumetinib PO (IUE only), (e) combination treatment with 30 mg/kg avapritinib and 1 mg/kg trametinib PO, or (f) combination treatment with 30 mg/kg avapritinib and 25 mg/kg selumetinib PO (IUE only). Mice were treated daily until morbidity.

Animals displaying symptoms of morbidity after treatment were euthanized for immunohistochemical or pharmacodynamic (PD) analysis of tumors. Mice were given an initial treatment dose for their assigned arm 1 hour before euthanasia. For immunohistochemical analysis, mice were perfused with Tyrode’s Solution followed by 4% paraformaldehyde fixative solution to preserve the structures of the brain.

### Multiplexed Immunofluorescence and Image Analysis of in vivo orthotopic tumor specimens

FFPE tissue sections were deparaffinized and rehydrated. Antigen retrieval was performed using a pressure cooker and 1x citrate buffer, pH 6.0. Subsequently, sections were stained using the Opal Polaris 7 Color Manual IHC Detection Kit (Akoya Biosciences). Image analysis was performed by using inForm analysis software (Akoya Biosciences) and the open-source software for digital pathology image analysis QuPath. The following primary antibodies were used at a 1:100 dilution: Recombinant Anti-Histone H3 (mutated K27M) antibody (Abcam, ab190631), Recombinant Anti-PDGFR alpha antibody (Abcam, ab234965), Purified Mouse Anti-Ki-67 Clone B56 (BD, 550609), Human Phospho-PDGFR alpha (Y762) Antibody (RnD Systems AF21141), Phospho-S6 Ribosomal Protein (Ser240/244) (D68F8) XP^®^ Rabbit mAb (Cell Signaling, 5364S), Phospho-Akt (Ser473) (736E11) Rabbit mAb (Cell Signaling, 3787) and EGFR Monoclonal Antibody H11 (Thermo Fisher, MA513070).

### Demographics and clinical monitoring of patients treated with avapritinib and MEK inhibitors

Five patients (n=3 pediatric, n=2 young adult) were treated with avapritinib and trametinib or avapritinib and selumetinib at a weight-based equivalent of the adult approved dose through compassionate use at the following institutions according to the expanded access compassionate-use program guidelines from Blueprint Medicines: University of Michigan (n=2), Nemours Children’s Health (n=1), Children’s Minnesota (n=1), Cleveland Clinic (n=1). Patients included n=1 CNS sarcoma, n=2 H3K27M DMG, and n=2 high-grade glioma cases. All patients harbored *PDGFRA* mutations. Laboratory parameters were regularly assessed, and magnetic resonance imaging (MRI) was conducted per clinician discretion to monitor tumor response and further analyzed by central radiological assessment on the basis of the Response Assessment in Pediatric Neuro-Oncology (RAPNO) criteria for pHGG and DIPG.^73,74^ Data was captured through an IRB-exempt protocol at the University of Michigan (HUM00262658).

### Tumor organoid culture

Through U of M IRB approved Tumor Bank and Koschmann lab Repository at the U of Michigan, patient-derived organoids (PDOs) were derived according to the Children’s Hospital of Philadelphia Standard Operating Procedure.^75^ Briefly, tissue from the operating room was carried on ice in a dissection media containing Hibernate A with 1% GlutaMax (100x) and 1% Antibiotic-Antimycotic (100x). Once arrived in the lab, in a biosafety cabinet, the media above the tissue was aspirated and the tissue was washed with DPBS++ (repeat 3x). Then, the tissue was put into a petri dish with new dissection media and micro dissected into 0.5–1mm pieces, with necrotic and hemorrhaged pieces removed. The dissected tissue pieces were washed again in DPBS++ in a conical tube (repeat 3x). The tissue pieces were then incubated with Red Blood Cell Lysis Buffer for 10 minutes at room temperature on a platform rocker and slowly rocked. The tissue pieces were then washed with DMEM/F-12 (+L-Glutamine, +2.438 g/L Sodium Bicarbonate) (repeat 3x). The tissue pieces were then equally transferred to a 6-well flat-bottom, ultra-low attached cell culture plate with 4mL of Glioblastoma Organoid Media and 1uL of 2-mercaptoetanol solution per well. The plate was then put on an orbital shaker rotating at 120 rpm inside a 37C, 5% CO2 incubator.

After growing in culture for 2–4 weeks, the PDOs were dissociated to single cell suspensions with Accumax, filtered through a 70 micron filter, and then checked for viability and cell count. The cells were appropriately seeded in 96-well plates and treated accordingly with FDA-approved drugs or drugs currently investigated in clinical trials. Agents were plated on our panel at a screening concentration of previously determined or predicted IC50 (half-maximal inhibitory concentration). Following the appropriate incubation period of 5–10 days, the Cell Titer Glo Assay was added to each well and the cells were assessed for viability. From this, a drug dose response figure was generated to display tumor response to each drug treatment.

### Quantification and Statistical Analysis

Statistical tests were performed using GraphPad Prism 10 or R 4.0.1. For cell and molecular biological methods, statistical analyses are described in detail in figure legends. For statistical comparisons within two groups, we used a two-tailed Student’s t-test. Significance values are given in the respective figures.

## Supplementary Material

1

## Figures and Tables

**Table 1:** Clinical identifiers associated with patient cases

Case	Initials	Patient ID	Treatment
Case 1	CD	UMPED191	Avapritinib/Trametinib
Case 2	SK	NEMOURS01	Avapritinib/Trametinib, Avapritinib/Selumetinib
Case 3	NC	UMPED144	Avapritinib/Trametinib
Case 4	BD	CHLDMN01	Avapritinib/Trametinib
Case 5	ZT	CLVCLN01	Avapritinib/Trametinib

**Table 2:** Adverse events associated with trametinib therapy in clinical cohort

Case #	ID	Adverse Event (Trametinib)	CTCAE Grade
1	UMPED191	Thrombocytopenia	3
		Neutropenia	2
		Acneiform rash	2
2	NEMOURS01	Acneiform rash	4
		Edema/hypoalbuminemia	3
3	UMPED144	Thrombocytopenia	2
		Diarrhea	1
4	CHLDMN01	Acneiform rash	3
		Musculoskeletal pain	2
		Oral ulcers	2
		Nausea	2
		Facial edema	1
5	CLVCLN01	Acneiform rash	3

## Statement of Significance:

High-grade glioma patients are in desperate need of improved treatment. The analyses in this study address weaknesses in current therapeutic strategies and propose improvements that would extend survival in this patient population.
