# Supplementary material for: Combinatorial Targeting of Avapritinib-Driven MAP Kinase Activation in High-Grade Glioma
Source: Res Sq. 2026 Jun 24:rs.3.rs-9974885. Preprint. [Version 1] doi: 10.21203/rs.3.rs-9974885/v1 (PMC13321252; doi:10.21203/rs.3.rs-9974885/v1)
Supplement: 1 [file NIHPPRS9974885V1-supplement-1.pdf]

Figure S1

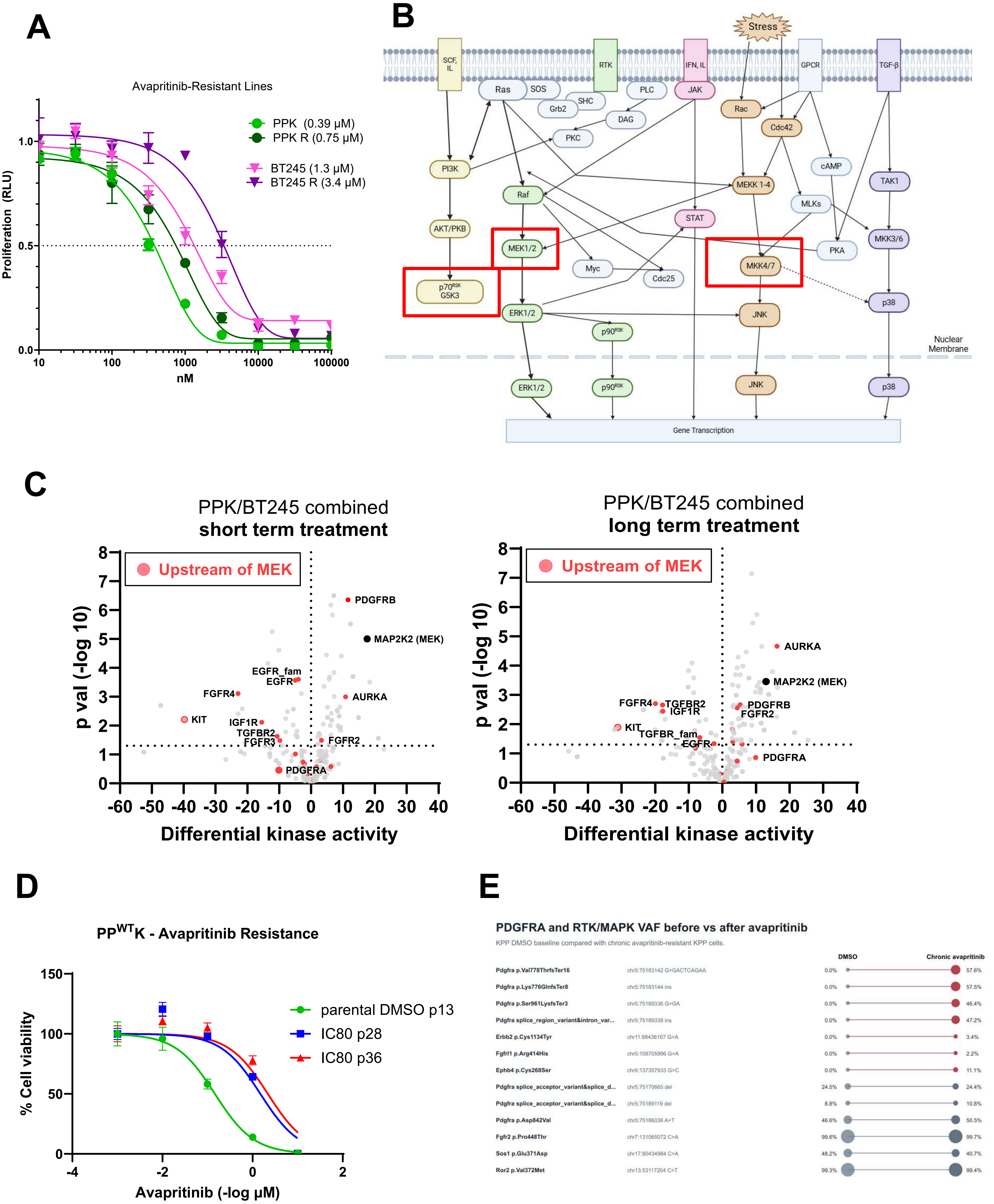
A
Avapritinib-Resistant Lines
PPK (0.39 μM)
PPK R (0.75 μM)
BT245 (1.3 μM)
BT245 R (3.4 μM)
Proliferation (RLU)
nM
B
Stress
SCF, IL
RTK
IFN, IL
GPCR
TGF-β
Ras
SOS
Grb2
SHC
PLC
JAK
DAG
PKC
Rac
Cdc42
PI3K
Raf
MEKK 1-4
cAMP
TAK1
MLKs
STAT
AKT/PKB
MEK1/2
PKA
MKK3/6
Myc
Cdc25
MKK4/7
G5K3
ERK1/2
JNK
p38
Nuclear Membrane
Gene Transcription
C
PPK/BT245 combined
short term treatment
Upstream of MEK
p val (-log 10)
Differential kinase activity
PDGFRB
MAP2K2 (MEK)
EGFR_fam
EGFR
FGFR4
AURKA
KIT
IGF1R
TGFBR2
FGFR3
FGFR2
PDGFRA
PPK/BT245 combined
long term treatment
Upstream of MEK
AURKA
MAP2K2 (MEK)
FGFR4
TGFBR2
IGF1R
PDGFRB
FGFR2
KIT
TGFBR_fam
EGFR
PDGFRA
D
PPWTK - Avapritinib Resistance
parental DMSO p13
IC80 p28
IC80 p36
% Cell viability
Avapritinib (-log μM)
E
PDGFRA and RTK/MAPK VAF before vs after avapritinib
KPP DMSO baseline compared with chronic avapritinib-resistant KPP cells.
DMSO
Chronic avapritinib
Pdgfra p.Val778ThrfsTer16
chr5:75183142 G>GACTCAGAA
0.0%
57.6%
Pdgfra p.Lys776GlnfsTer8
chr5:75183144 ins
0.0%
57.5%
Pdgfra p.Ser961LysfsTer3
chr5:75189336 G>GA
0.0%
46.4%
Pdgfra splice_region_variant&intron_var...
chr5:75189338 ins
0.0%
47.2%
Erbb2 p.Cys1134Tyr
chr11:98436167 G>A
0.0%
3.4%
Fgfrl1 p.Arg414His
chr5:108705996 G>A
0.0%
2.2%
Ephb4 p.Cys268Ser
chr5:137357933 G>C
0.0%
11.1%
Pdgfra splice_acceptor_variant&splice_d...
chr5:75170665 del
24.5%
24.4%
Pdgfra splice_acceptor_variant&splice_d...
chr5:75189119 del
8.8%
10.8%
Pdgfra p.Asp842Val
chr5:75186036 A>T
46.6%
50.5%
Fgfr2 p.Pro448Thr
chr7:131065072 C>A
99.6%
99.7%
Sos1 p.Glu371Asp
chr17:80434984 C>A
48.2%
40.7%
Ror2 p.Val372Met
chr13:53117204 C>T
99.3%
99.4%

Figure S1: Supportive data from kinome analysis

(**A**) 72-hour dose-response curves of resistant and parent cell models to avapritinib.

(**B**) Diagram of MAPK signaling pathway.

(**C**) HT-KAM data from Fig. 1C, highlighting receptor tyrosine kinases and other proteins found to be upstream of the Raf/Mek/Erk pathway.

(**D**) Dose-response curves of parental and resistant PPK* cells treated with avapritinib.

(**E**) Dot plot demonstrating *PDGFRA* variants that arose after chronic avapritinib treatment in PPK* cells.

Figure S2

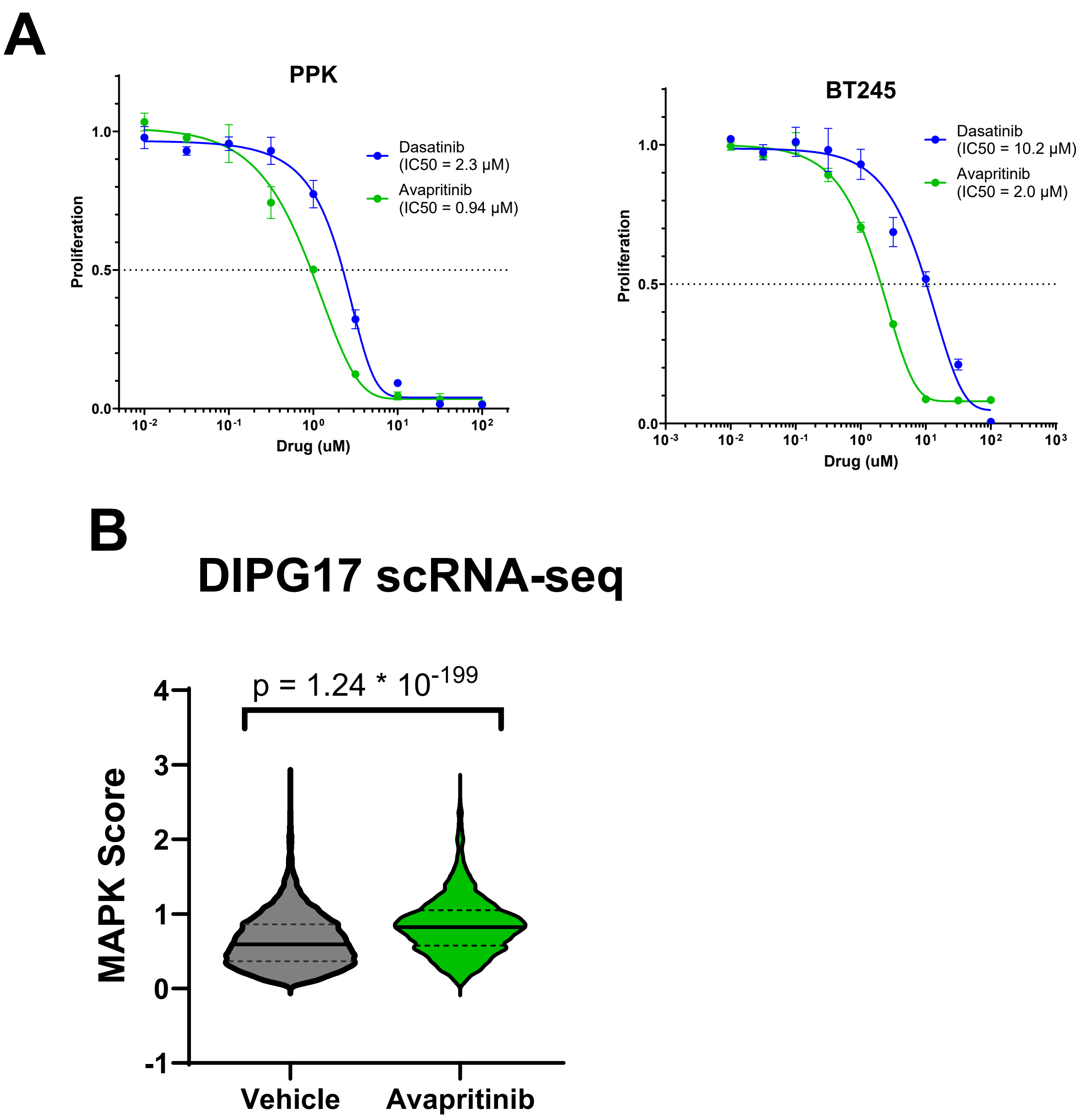
A
PPK
Dasatinib
(IC50 = 2.3 μM)
Avapritinib
(IC50 = 0.94 μM)
Proliferation
Drug (uM)
BT245
Dasatinib
(IC50 = 10.2 μM)
Avapritinib
(IC50 = 2.0 μM)
Proliferation
Drug (uM)
B
DIPG17 scRNA-seq
p = 1.24 * 10-199
MAPK Score
Vehicle
Avapritinib

Figure S2: Supportive data on avapritinib-induced sustained MAPK activity

(**A**) 72-hour dose response curves of PPK and BT245 cells to dasatinib and avapritinib.

(**B**) Overall MAPK score from scRNA-seq results.

Figure S3

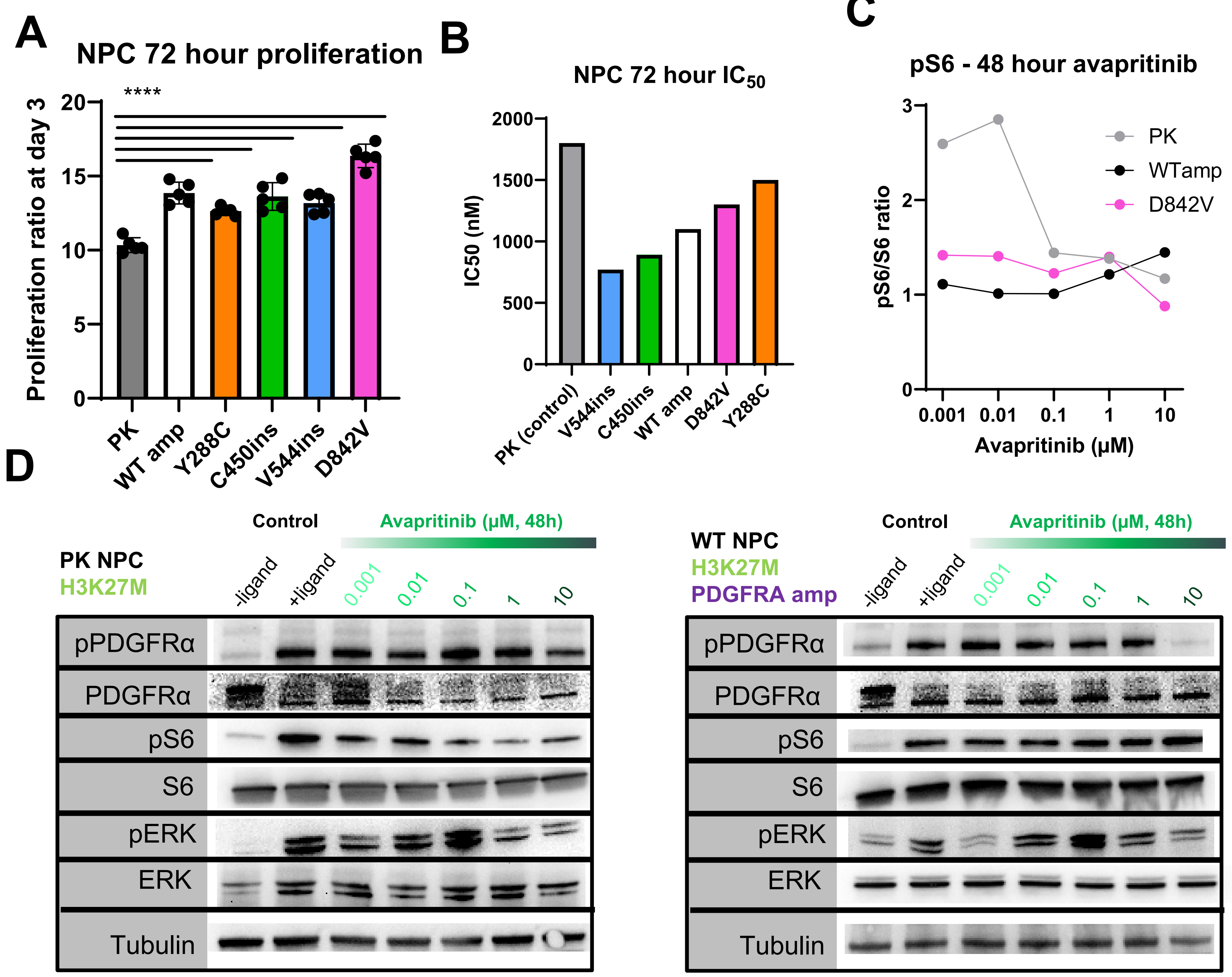
A
NPC 72 hour proliferation
****
Proliferation ratio at day 3
20
15
10
5
0
PK
WT amp
Y288C
C450ins
V544ins
D842V
B
NPC 72 hour IC50
IC50 (nM)
2000
1500
1000
500
0
PK (control)
V544ins
C450ins
WT amp
D842V
Y288C
C
pS6 - 48 hour avapritinib
pS6/S6 ratio
3
2
1
0
PK
WTamp
D842V
0.001
0.01
0.1
1
10
Avapritinib (μM)
D
Control
Avapritinib (μM, 48h)
PK NPC
H3K27M
-ligand
+ligand
0.001
0.01
0.1
1
10
pPDGFRα
PDGFRα
pS6
S6
pERK
ERK
Tubulin
WT NPC
H3K27M
PDGFRA amp
Control
Avapritinib (μM, 48h)
-ligand
+ligand
0.001
0.01
0.1
1
10
pPDGFRα
PDGFRα
pS6
S6
pERK
ERK
Tubulin

Figure S3: Supportive data on impact of PDGFRA variant type on avapritinib-induced MAPK activity

(**A**) Proliferation ratio of NPCs at 72 hours of growth, normalized to hour 0 for each variant.

(**B**) IC50 results of 72-hour avapritinib dose response curve of NPC variants.

(**C**) Quantification of pS6 Western blots in **Figure 3E**, normalized to control.

(**D**) 48-hour Western blot of non-PDGFRA-altered (PK) and PDGFRA wildtype amplified (WT) NPCs, examining phosphorylated PDGFRA, ERK, and S6 expression.

Figure S4

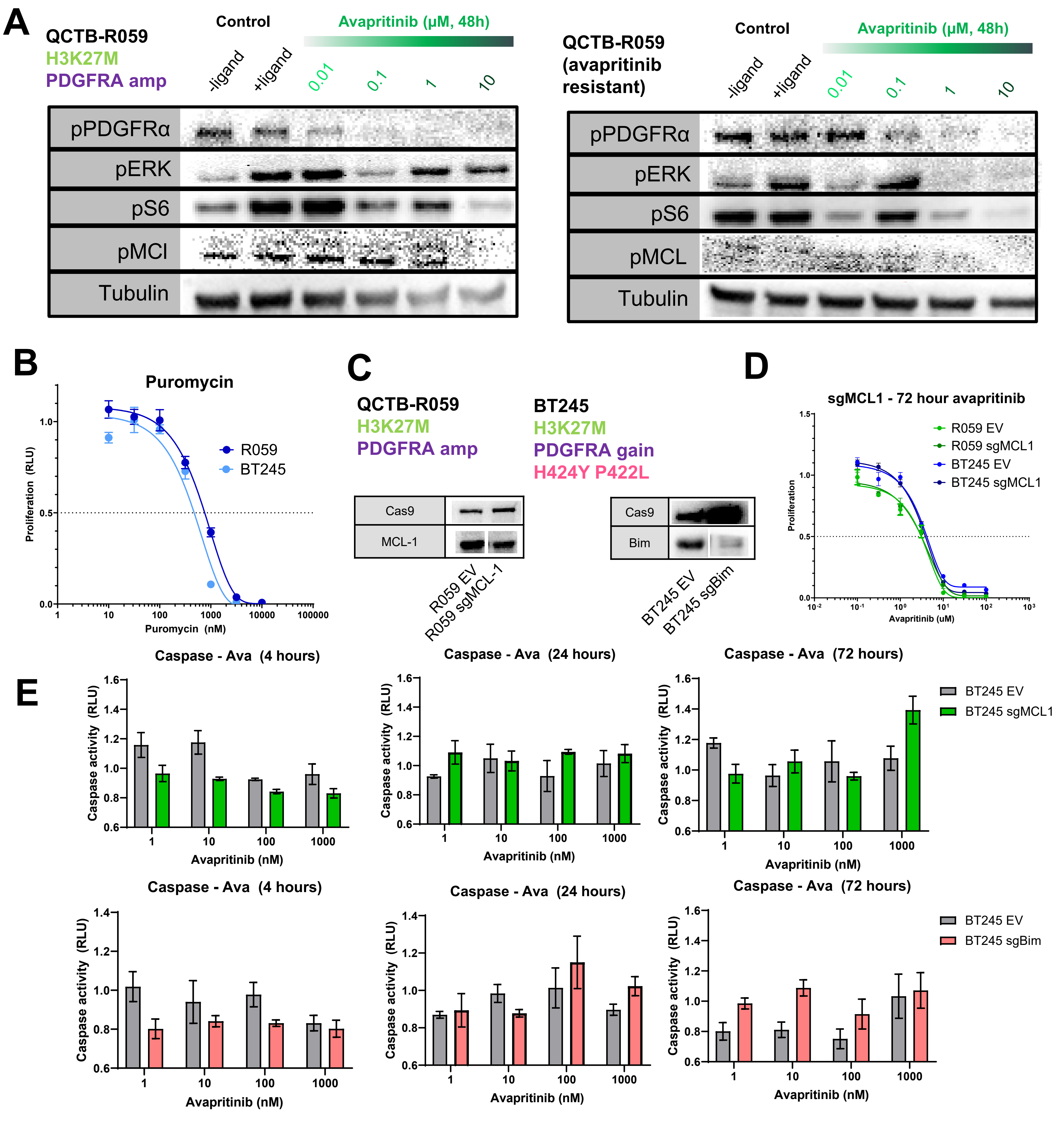

A
QCTB-R059
H3K27M
PDGFRA amp
Control
Avapritinib (μM, 48h)
-ligand
+ligand
0.01
0.1
1
10
pPDGFRα
pERK
pS6
pMCl
Tubulin
QCTB-R059 (avapritinib resistant)
pMCL
B
Puromycin
R059
BT245
Proliferation (RLU)
Puromycin (nM)
C
QCTB-R059
H3K27M
PDGFRA amp
BT245
H3K27M
PDGFRA gain
H424Y P422L
Cas9
MCL-1
R059 EV
R059 sgMCL-1
Bim
BT245 EV
BT245 sgBim
D
sgMCL1 - 72 hour avapritinib
R059 EV
R059 sgMCL1
BT245 EV
BT245 sgMCL1
Proliferation
Avapritinib (uM)
E
Caspase - Ava (4 hours)
Caspase - Ava (24 hours)
Caspase - Ava (72 hours)
Caspase activity (RLU)
Avapritinib (nM)
BT245 EV
BT245 sgMCL1
BT245 sgBim

Figure S4: Supportive data on MAPK activity and avapritinib-induced apoptosis

(**A**) Western blot of parent and resistant R059 cells after 48 hours of avapritinib exposure.

(**B**) Puromycin dose response-curves of parent cell lines transduced via lentiCRISPR.

(**C**) Confirmatory Western blot of Cas-9 expression and MCL-1 and Bim knockout in lentiCRISPR-transduced pHGG cell models.

(**D**) 72-hour dose response curves to avapritinib of QCTB-R059 and BT245, transfected with empty vector and sgMCL-1 lentiCRISPR-v2 plasmids.

(**E**) Apoptosis levels as measured by CaspaseGlo assay at varying time points and concentrations, both with sgMCL-1 and sgBim.

Figure S5

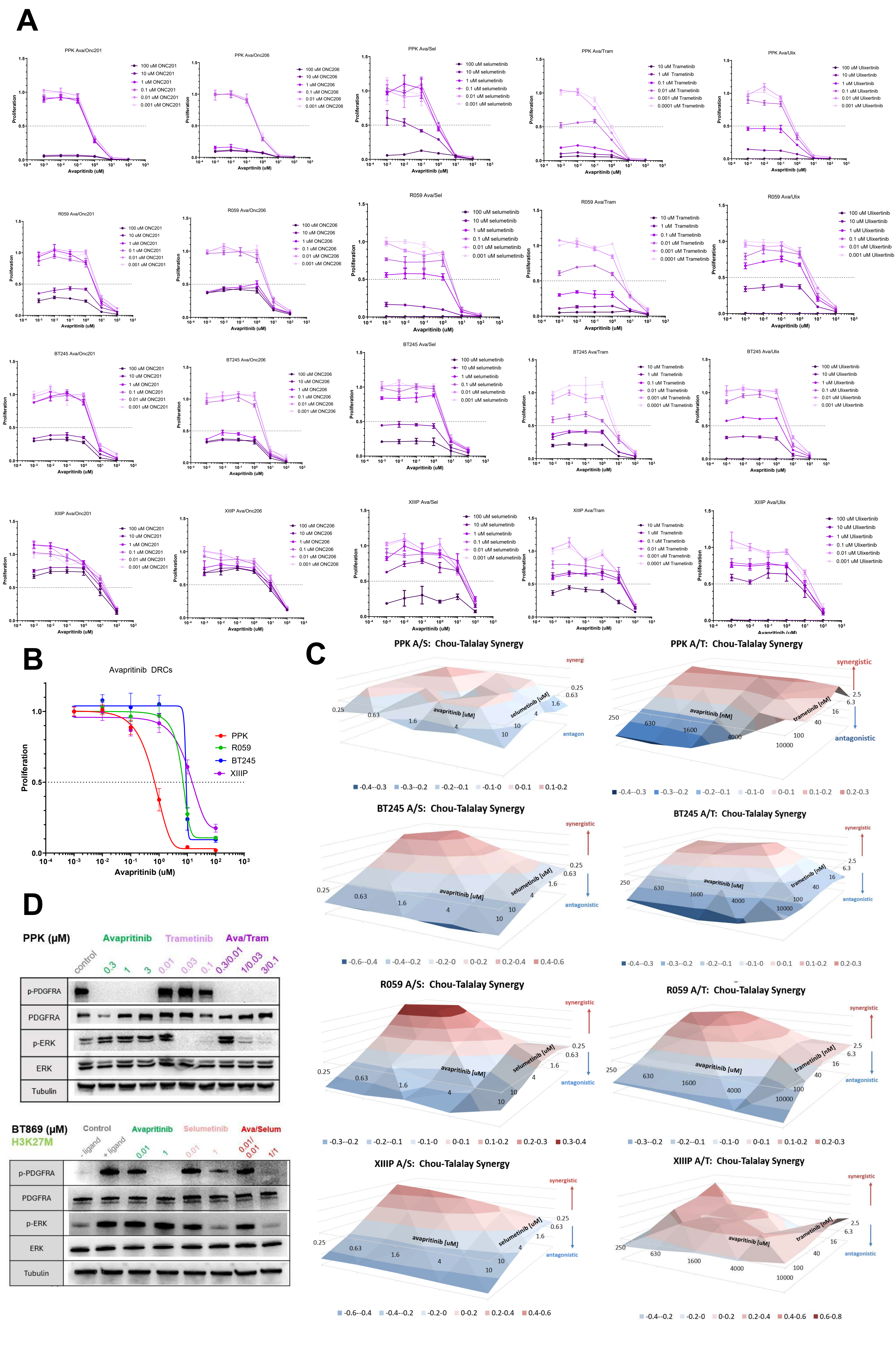
A
B
C
D
Avapritinib DRCs
PPK
R059
BT245
XIIIP
Proliferation
Avapritinib (uM)
PPK A/S: Chou-Talalay Synergy
PPK A/T: Chou-Talalay Synergy
BT245 A/S: Chou-Talalay Synergy
BT245 A/T: Chou-Talalay Synergy
R059 A/S: Chou-Talalay Synergy
R059 A/T: Chou-Talalay Synergy
XIIIP A/S: Chou-Talalay Synergy
XIIIP A/T: Chou-Talalay Synergy
synergistic
antagonistic
PPK (μM)
Avapritinib
Trametinib
Ava/Tram
control
p-PDGFRA
PDGFRA
p-ERK
ERK
Tubulin
BT869 (μM)
H3K27M
Control
Selumetinib
Ava/Selum
- ligand
+ ligand

Figure S5: Supportive data on PDGFRα/MAPK targeting

(**A**) Combinatorial therapy dose response curves of avapritinib and MAPK inhibitor; all concentrations of avapritinib are visualized.

(**B**) 72-hour dose response curves to avapritinib monotherapy for all models tested.

(**C**) Additional Chou-Talalay synergism studies between avapritinib and MEK inhibitor selumetinib or trametinib using multiple HGG cell models.

(**D**) Additional Western blots of various cell models treated with avapritinib, trametinib or selumetinib, and combination.

Figure S6

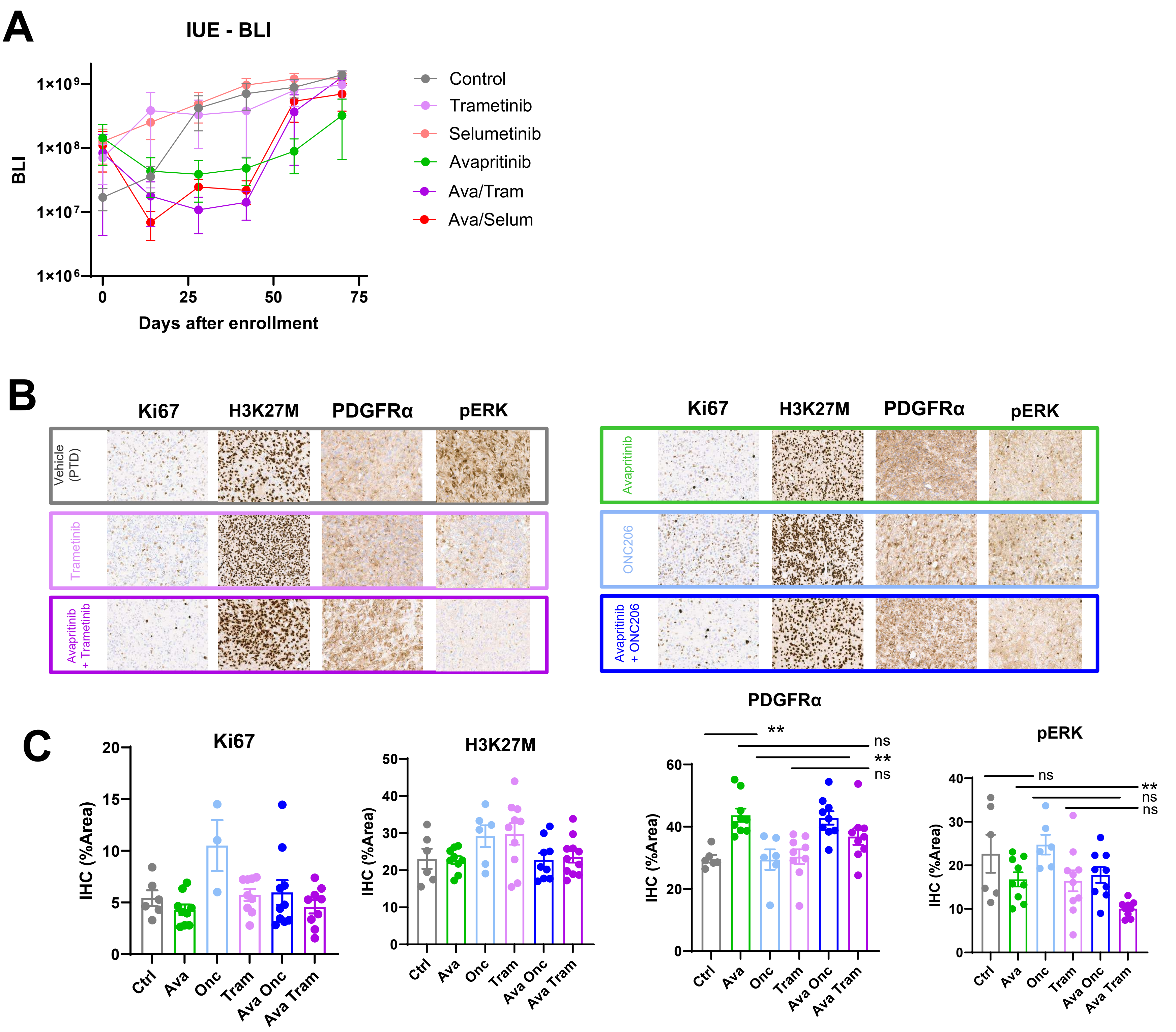

A
IUE - BLI
BLI
1×10⁹
1×10⁸
1×10⁷
1×10⁶
0
25
50
75
Days after enrollment
Control
Trametinib
Selumetinib
Avapritinib
Ava/Tram
Ava/Selum
B
Ki67
H3K27M
PDGFRα
pERK
Vehicle (PTD)
Trametinib
Avapritinib + Trametinib
Avapritinib
ONC206
Avapritinib + ONC206
C
Ki67
H3K27M
PDGFRα
pERK
IHC (%Area)
Ctrl
Ava
Onc
Tram
Ava Onc
Ava Tram
**
ns
ns

Figure S6: Supportive data on combinatorial therapy *in vivo*

(**A**) Luminescence of IUE treatment study plotted over time.

(**B**) IHC results and (**C**) quantification of Ki67, H3K27M, PDGFRα, and pERK stains from PPK orthotopic treatment experiment.

Figure S7

A

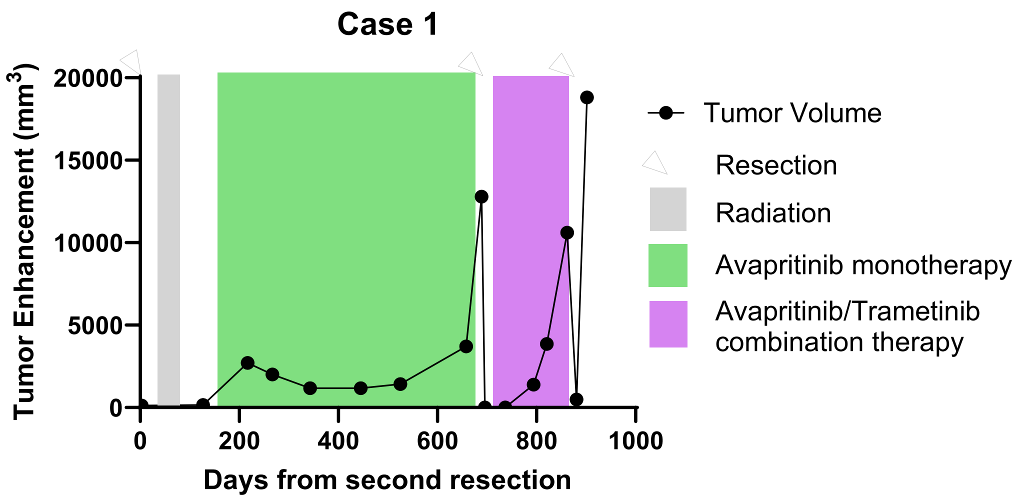

Case 1
Tumor Enhancement (mm³)
20000
15000
10000
5000
0
0
200
400
600
800
1000
Days from second resection
Tumor Volume
Resection
Radiation
Avapritinib monotherapy
Avapritinib/Trametinib combination therapy

B

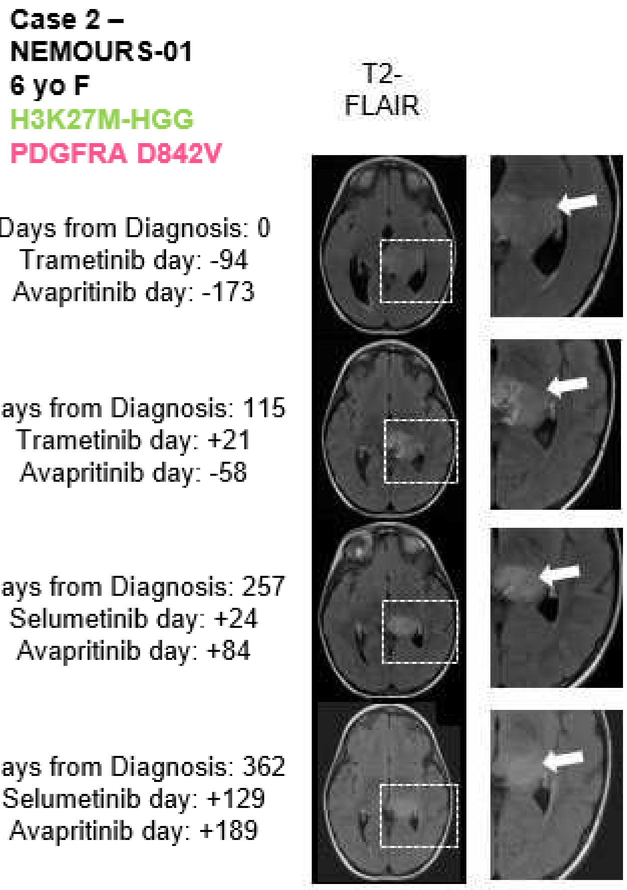

Case 2 – NEMOURS-01
6 yo F
H3K27M-HGG
PDGFRA D842V
T2-FLAIR
Days from Diagnosis: 0
Trametinib day: -94
Avapritinib day: -173
Days from Diagnosis: 115
Trametinib day: +21
Avapritinib day: -58
Days from Diagnosis: 257
Selumetinib day: +24
Avapritinib day: +84
Days from Diagnosis: 362
Selumetinib day: +129
Avapritinib day: +189

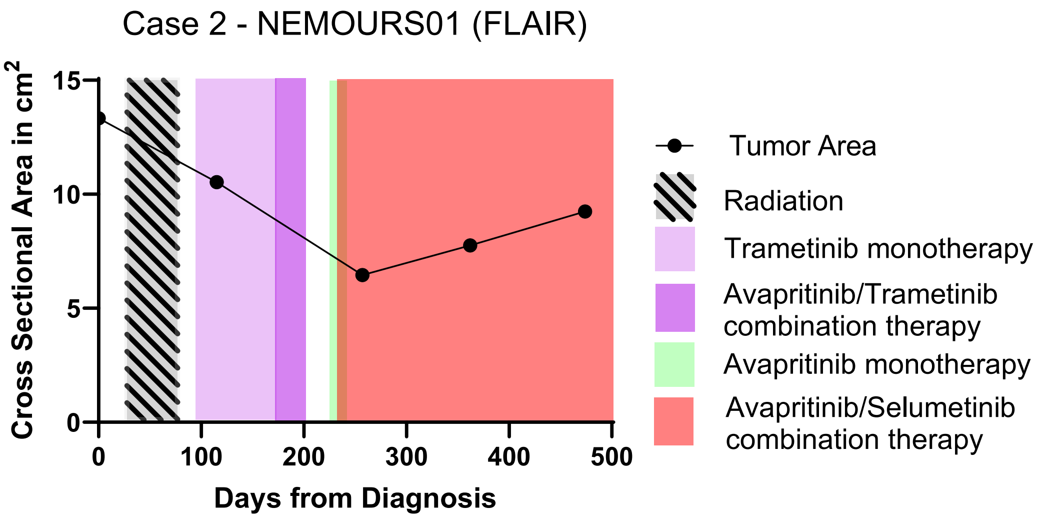

Case 2 - NEMOURS01 (FLAIR)
Cross Sectional Area in cm²
15
10
5
0
0
100
200
300
400
500
Days from Diagnosis
Tumor Area
Radiation
Trametinib monotherapy
Avapritinib/Trametinib combination therapy
Avapritinib monotherapy
Avapritinib/Selumetinib combination therapy

Figure S7: Supportive data on clinical combinatorial data

(**A**) Tumor volume over time for Case 1 (UMPED191).

(**B**) Representative FLAIR images and tumor volume over time for Case 2 (NEMOURS-01).
